# Oligodendroglia Generated From Adult Rat Adipose Tissue by Direct Cell Conversion

**DOI:** 10.3389/fcell.2022.741499

**Published:** 2022-02-11

**Authors:** Lara Vellosillo, Jorge Pascual-Guerra, Maria Paz Muñoz, José Antonio Rodríguez-Navarro, Daniel González-Nieto, Luis Carlos Barrio, Maria del Val Toledo Lobo, Carlos Luis Paíno

**Affiliations:** ^1^ Servicio de Neurobiología-Investigación, IRYCIS, Hospital Universitario Ramón y Cajal, Madrid, Spain; ^2^ Center for Biomedical Technology (CTB), Universidad Politécnica, Madrid, Spain; ^3^ Departamento de Biología Celular, Universidad Complutense, Madrid, Spain; ^4^ Unidad de Neurología Experimental, IRYCIS, Hospital Universitario Ramón y Cajal, Madrid, Spain; ^5^ Departamento de Biomedicina y Biotecnología, IRYCIS, Universidad de Alcalá, Alcalá de Henares, Spain

**Keywords:** ADSC, oligodendrocyte precursor cells, myelination, benztropine, adult rat, direct lineage conversion

## Abstract

Obtaining oligodendroglial cells from dispensable tissues would be of great interest for autologous or immunocompatible cell replacement therapy in demyelinating diseases, as well as for studying myelin-related pathologies or testing therapeutic approaches in culture. We evaluated the feasibility of generating oligodendrocyte precursor cells (OPCs) from adult rat adipose tissue by expressing genes encoding transcription factors involved in oligodendroglial development. Adipose-derived mesenchymal cells were lentivirally transduced with tetracycline-inducible *Sox10*, *Olig2*, *Zfp536*, and/or *Nkx6.1* transgenes. Immunostaining with the OPC-specific O4 monoclonal antibody was used to mark oligodendroglial induction. O4- and myelin-associated glycoprotein (MAG)-positive cells emerged after 3 weeks when using the *Sox10* + *Olig2* + *Zfp536* combination, followed in the ensuing weeks by GFAP-, O1 antigen-, p75NTR (low-affinity NGF receptor)-, and myelin proteins-positive cells. The O4^+^ cell population progressively expanded, eventually constituting more than 70% of cells in culture by 5 months. *Sox10* transgene expression was essential for generating O4^+^ cells but was insufficient for inducing a full oligodendroglial phenotype. Converted cells required continuous transgene expression to maintain their glial phenotype. Some vestigial characteristics of mesenchymal cells were maintained after conversion. Growth factor withdrawal and triiodothyronine (T_3_) supplementation generated mature oligodendroglial phenotypes, while FBS supplementation produced GFAP^+^- and p75NTR^+^-rich cultures. Converted cells also showed functional characteristics of neural-derived OPCs, such as the expression of AMPA, NMDA, kainate, and dopaminergic receptors, as well as similar metabolic responses to differentiation-inducing drugs. When co-cultured with rat dorsal root ganglion neurons, the converted cells differentiated and ensheathed multiple axons. We propose that functional oligodendroglia can be efficiently generated from adult rat mesenchymal cells by direct phenotypic conversion.

## 1 Introduction

Oligodendrocyte transplantation has long been proposed to be a feasible strategy for repairing demyelinated lesions (Blakemore and Franklin, 1991; Archer et al., 1997; Wang et al., 2013), an approach that would be facilitated by the use of autologous or immunocompatible cells. Moreover, cultures of oligodendroglia from patients suffering genetic leukodystrophies would allow studying the basis of their impaired myelination as well as testing therapeutic approaches in a dish, aiming to identify personalized treatments. However, there are obvious limitations to obtaining oligodendrocytes from the central nervous system (CNS) of patients or donors. Consequently, it would be of great interest to identify alternative means of generating oligodendroglia from the peripheral tissues of patients.

Several procedures for obtaining expandable and myelinogenic oligodendrocytes from pluripotent stem cells (PSCs) in culture have been reported (Keirstead et al., 2005; Izrael et al., 2007; Hu et al., 2009; Najm et al., 2011; Douvaras et al., 2014; Douvaras and Fossati, 2015; Piao et al., 2015), including protocols that can promote the differentiation of oligodendrocytes from induced PSCs (iPSCs), themselves generated through the reprogramming of adult somatic cells (Wang et al., 2013; Douvaras et al., 2014; Douvaras and Fossati, 2015). More recently, some groups have proposed the rapid generation of oligodendroglia from human iPSCs *via* the exogenous expression of a combination of *Sox10*, *Olig2*, and *Nkx6.2* (Ehrlich et al., 2017) or only *Sox10* (Garcia-Leon et al., 2018). This process involves three steps, namely, the generation, selection, characterization, and the growing of iPSCs from cultures of adult somatic cells; deriving neural progenitor cells from the iPSCs; and using transduction to express genes encoding oligodendroglial transcription factors in these cells. However, in addition to the protracted time needed for cell reprogramming, the risk remains that, unless all cells are differentiated, residual PSCs might continue to proliferate and produce teratomas.

Two laboratories have simultaneously reported that embryonic rat or mouse fibroblasts can be directly converted into oligodendroglial cells through the exogenous expression of genes coding for transcription factors involved in oligodendrocyte development (Najm et al., 2013; Yang et al., 2013). Furthermore, these cells were shown to be capable of myelinating axons both *in vivo* and in culture. However, cell replacement therapies, as well as the search for personalized treatment strategies, would require the use of adult somatic cells, but evidence that these procedures can be used to directly convert cells from adult somatic tissues into oligodendrocytes is lacking.

The vasculo–stromal fraction of adipose tissue contains mesenchymal stem cells (MSCs) that can be easily grown in culture, and thus represents an accessible source of mesenchymal cells. These cells display characteristics such as immunomodulatory (Bai et al., 2009; Fransson et al., 2014; Ma et al., 2014; Mattar and Bieback, 2015), anti-inflammatory (Marfia et al., 2016; van Velthoven et al., 2017), neuroprotective (Otero-Ortega et al., 2015; Ribeiro et al., 2015), and trophic properties (Bai et al., 2012; Drago et al., 2013; Pires et al., 2016), rendering them potentially suitable for use in the treatment of demyelinating diseases.

Here, we show that cultured mesenchymal cells from adult rats can be directly converted into induced oligodendroglia-like cells as well as other macroglial cell types through the expression of three transgenes coding for transcription factors involved in oligodendroglial development. We further demonstrate that these induced cells show a repertoire of molecular traits similar to those of, and respond to the same pharmacological cues as, neural-derived oligodendroglia.

## 2 Materials and Methods

A list of commercial product references is provided in Supplementary Table S1.

### 2.1 Animals and Procedures

All procedures involving animals were performed by qualified personnel and in accordance with Directive 2010/63/UE of the European Union on the protection of animals used for scientific purposes and its transposition to Spanish law (RD53/2013). Rats were bred at the animal facilities of the Hospital Universitario Ramón y Cajal (ES280790002001). For tissue collection, the rats were first deeply anesthetized with isoflurane and subsequently euthanized by decapitation. Ethics committee approval was not required for these procedures.

### 2.2 Adipose Tissue-Derived Mesenchymal Stem Cells Culture

Adipose tissue was dissected under aseptic conditions from the inguinal pads of 16 Sprague–Dawley rats weighing 220–250 g. The tissue was cleaned of fasciae and major blood vessels, cut into 1-mm^2^ pieces, and digested for 40 min at 37°C with 1 mg/ml collagenase A in αMEM supplemented with 20% FBS, non-essential amino acids, glutamine, and antibiotics/antimitotics (Gibco; henceforth referred to as α20 medium). DNAse I (20 μg/ml) was added to prevent cell clumping due to DNA released from dead cells and the tissue was then dispersed by repeated passage through a P1000 automatic pipette using sterile filter tips. Large, undispersed clumps of tissue were removed by passing the suspension through 100-μm mesh cell strainers. After centrifugation at 400 ×g for 3 min, the supernatant, including the layer of floating adipose cells, was discarded. The pellet was resuspended in 1 ml of α20 medium and seeded in a T75 flask (Falcon) containing the same medium. The next day, the medium with unattached cells was removed, the flask was rinsed once with Hank’s balanced salt solution (HBSS), and fresh α20 medium was added. These adipose-derived stromal cells (ADSCs) proliferated and reached near-confluence after 4–5 days, at which point they were detached with 0.05% trypsin + 0.02% EDTA, centrifuged, resuspended in α20 medium, and quantified using a hemocytometer. The cells were then seeded in new T flasks at 660 cells/cm^2^ in α20 medium. The medium was changed on day 4 and the cells were passaged on day 7, at which time they were again confluent, their number having increased 30–40-fold. After 4 passages, the cultures showed signs of cellular senescence and slow proliferation. Consequently, the experiments were performed using cells at passages 1–3.

The mesenchymal characteristics of these cells were tested by assessing their adipogenic, chondrogenic, and osteogenic differentiation potential (Pittenger et al., 1999) using standard procedures.

### 2.3 Lentiviral Particle Production

Self-inactivating, replication-incompetent lentiviruses were produced in HEK293T cells by co-transfecting the lentiviral plasmid (see below), the packaging plasmid psPAX2, and the envelope vector pCMV-VSV-G using Lipofectamine 2000. The cells were maintained in Dulbecco’s minimal essential medium (DMEM) supplemented with 10% FBS. Culture supernatants were collected every 24 h for 3 days, pooled, centrifuged at 1,000 **×**g for 10 min, passed through sterile 0.45-µm filters, layered over 2 ml of 20% sterile sucrose, pelleted by ultracentrifugation at 50,000 **×**g for 3 h at 4°C, resuspended in 500 µL of DMEM, and aliquoted.

Viral titers were measured with the Lentivirus qPCR kit (Applied Biological Materials; contained reverse transcriptase, standards, and primers specific for the lentiviral 5′-LTR) using LightCycler 480 with SybrGreen I Master kit (Roche Applied Science). Titers ranged between 120,000 and 500,000 lentiviral particles per µL. Viral particle-to-cell ratios of 1:1, 5:1, and 10:1 were tested for ADSC transduction.

### 2.4 Plasmids

The FUW-M2rtTA plasmid was a gift from Rudolf Jaenisch (Addgene plasmid # 20342) (Hockemeyer et al., 2008); Tet-O-FUW-Sox10 (Addgene plasmid # 45843), Tet-O-FUW-Zfp536 (Addgene plasmid # 45845), Tet-O-FUW-Nkx6.1 (Addgene plasmid # 45846), Tet-O-FUW-Olig2 (Addgene plasmid # 30131), and Tet-O-FUW-EGFP (Addgene plasmid # 30130) were gifts from Marius Wernig (Vierbuchen et al., 2010; Yang et al., 2013); psPAX2 was a gift from Didier Trono (Addgene plasmid # 12260); and pCMV-VSV-G was a gift from Bob Weinberg (Addgene plasmid # 8454) (Steward et al., 2006).

### 2.5 Lentiviral Transduction

MSCs were seeded at 15,000 cells/cm^2^ in 24-well plates and allowed to grow for 24 h in α20 medium. Viral particles and 8 μg/ml polybrene were added to the culture medium and the plates were incubated at 37°C for 1 h in an Eppendorf centrifuge at 1,000 **×**g and then for 1 h in an incubator with 5% CO_2_, following which the medium was replaced with fresh α20 medium. After 24 h, the cells were detached with trypsin (as above) and seeded in new flasks or plates for expansion or experimental analysis. Where appropriate, after transfection, cells were cryopreserved at −80°C in FBS with 10% DMSO.

To prevent cells from receiving multiple transgene doses, lentiviral titers were limited to that necessary for transducing approximately 30% of cells in each culture. Transduction efficiency was assessed by analysis of SOX10 immunofluorescence at different time points following the doxycycline-mediated activation of transgene expression. Four days after ADSC transduction with the *Sox10* + *Olig2* + *Zfp536* (+M2rtTA) transgenes (subsequently referred to as S + O + Z-transduced cells), an average of 8.9% of cells showed nuclear labeling for Sox10. After 12 days, 38.9% of the transduced cells were Sox10^+^, and the cell numbers had increased by 496% relative to control cultures transduced with M2rtTA only. After 45 days, 53.7% of the cells were Sox10^+^, and after 100 days, nearly all the cells showed intense nuclear Sox10 staining. These observations indicated that cells expressing the *Sox10* transgene were proliferating and that the proportion of Sox10^+^ cells increased when cultures were maintained in neurobasal medium with B27 supplement (NBB27 medium) (Invitrogen) containing EGF + basic FGF (bFGF) + PDGF-AA + doxycycline (see below). Additionally, the low numbers of Sox10^+^ cells observed at day 4 (cells that had been simultaneously transduced with the *Sox10* and the M2rtTA transgenes) were nevertheless sufficient to generate pure cell cultures expressing Sox10 in 3 months or less because of their selective proliferation in the present medium conditions.

### 2.6 Culture Medium for Transduced Cells

After transduction, the cells were cultured in NBB27 medium. To induce transgene expression, doxycycline (1 μg/ml) was added to the growth medium. For OPC growth, NBB27 was supplemented with EGF (20 ng/ml), bFGF (20 ng/ml), PDGF-AA (10 ng/ml), d-biotin (10 ng/ml), and doxycycline hydrochloride (OPC medium). To increase the efficiency of oligodendroglial conversion, several pre-treatments were tested (Supplementary Figure S6; Supplementary Table S2). This included attempts to neuralize the cells by exposure to 1 mM all-*trans* retinoic acid (RA) for 5 days, to prevent ADSCs from adopting a chondrogenic phenotype when Sox10 was expressed (as suggested in Supplementary Figure S2) (Chimal-Monroy et al., 2003) by incubating in adipogenic differentiation cocktail (see below) for 5 days, or to treat with 25 nM Repsox before the initial doxycycline exposure. The adipocyte pre-differentiation cocktail initially included 10 μg/ml insulin, 1 µM dexamethasone, 200 µM indomethacin, and 250 µM 3-isobutyl-1-methylxanthine (IBMX), but this was later reduced to indomethacin + IBMX to improve cell adhesion to the culture surface.

### 2.7 Immunocytochemistry

Cells were cultured on glass coverslips in the wells of 24-well plates for at least 48 h, and then fixed in buffered 4% paraformaldehyde for 10 min and washed in PBS, pH 7.4. To detect surface antigens, after blocking with 5% normal goat serum in PBS, cells were incubated with antibodies targeting O4 sulfatide, O1/galactocerebroside (GalC), NG2, p75NTR or A2B5 without permeabilization for 90 min at room temperature. For the immunostaining of intracellular antigens, cells were post-fixed and permeabilized with ethanol: acetic acid (19:1) for 10 min at −20°C and then incubated with the respective primary antibodies overnight at 4°C. The next day, the cells were incubated with anti-mouse, anti-rat, or anti-rabbit antibodies conjugated to Alexa fluorochromes (1:500 dilution) for 30–45 min at room temperature in the dark. Both primary and secondary antibodies were diluted in blocking solution. Nuclei were counterstained with bisBenzimide hydrochloride (Hoechst 33342, 3 × 10^–5^ M) in PBS for 5 min. The coverslips were rinsed in distilled water and mounted on slides with Prolong Gold. The samples were analyzed and imaged using an Olympus BX51 fluorescence microscope or a Nikon Eclipse Ti confocal microscope. The list of antibodies and suppliers is shown in Supplementary Table S5.

### 2.8 RT-PCR

Total RNA was isolated from transduced cells, control ADSCs, or neural tissue-derived oligospheres using the RNeasy Mini Kit or the GeneJET RNA Purification Kit. RNA was reverse transcribed using the First-Strand RNA Synthesis Kit and PCR was performed in a thermal cycler (Applied Biosystems) using a reaction mixture containing specific primers, dNTPs, and AmpliTaq DNA polymerase. Amplified cDNAs were electrophoretically separated in 1.5%–2% agarose gels loaded with the fluorochrome GreenSafe Premium. Bands were documented and analyzed using Molecular Imager Gel Doc (Bio-Rad).

For RT-qPCR, 1,000 ng of total RNA was reverse transcribed using the NZY First-Strand cDNA Synthesis Kit. Real-time PCR was performed using TB Green Premix Ex Taq (Takara Bio) or LightCycler 480 II reagents (Roche Applied Science). The initial denaturation step was 95°C for 5 min, followed by 45 cycles at 95°C for 5 s and 60°C for 30 s (for Takara reagents) or 95°C for 10 s, 60°C for 15 s, and 72°C for 15 s (when using LightCycler reagents). The melting curves for the PCR products were evaluated at the end of the amplification reactions. The final PCR reaction products were separated on 2% agarose gels containing GreenSafe Premium to confirm the presence of a single band. The efficiency of the reaction for each primer pair was calibrated by amplifying serial dilutions (1:10, 1:100, 1:1,000, and 1:10,000) of cDNA from positive controls. The association between the threshold cycle (Ct) and the log[RNA] was linear (−3.45 < slope < −3.32). The relative expression levels of target genes were normalized to that of the housekeeping gene glyceraldehyde 3-phosphate dehydrogenase (*Gapdh*) using the ΔCt method (Pfaffl, 2001). For quantification, cDNA samples were diluted 1/10 in the reaction mix. The primers used in real-time and end-point PCR are shown in Supplementary Tables S3 and S4.

### 2.9 Cultures of Neural Tissue-Derived Oligospheres

Control oligodendroglia-rich cultures were generated from the cervical spinal cords of E16 rats (the same animals from which the dorsal root ganglia were obtained; see below) or the cerebral cortices of 3–6-day-old postnatal rat pups. In both cases, the neural tissue, free of meninges, was mechanically dispersed by passing through a P1000 automatic pipette fitted with a fire-smoothened sterile filter tip, following which the cell suspension was passed through a 100-µm mesh cell strainer and seeded directly in flasks in NBB27 medium supplemented with EGF (20 ng/ml) and bFGF (20 ng/ml). The cell suspension was collected and pelleted by centrifugation at 400 ×g for 3 min and seeded in a new flask every day for the first 3 days to discard adherent cells. To produce single-cell suspensions and to eliminate cell debris without the need for gradient centrifugation, samples were digested with accutase first for 10 min at room temperature and then for 10 min at 37°C, followed by trituration with fire-narrowed P1000 sterile filter tips. These cultures were transferred to new flasks and provided with fresh NBB27 medium supplemented with EGF + bFGF every 2**–**3 days. Numerous medium-sized neurospheres (i.e., cell aggregates rich in neural progenitors) had formed by day 10. Thereafter, the medium was additionally supplemented with 10 ng/ml PDGF-AA to promote the oligodendroglial commitment of neural progenitors, eventually leading to the generation of “oligospheres,” floating aggregates highly enriched in oligodendroglial cells (58%**–**95% O4^+^ cells and ∼90% NG2^+^ cells; see Supplementary Figure S14). For the seeding of these cells on adherent surfaces (poly-L-ornithine-coated flasks, wells, or coverslips), the oligospheres were digested with accutase and disaggregated mechanically as described above. The seeded cells were maintained in NBB27 medium supplemented with either EFG + bFGF + PDGF-AA, for OPCs, or triiodothyronine (T_3_), for differentiated oligodendroglia showing high O1 positivity (as shown in Supplementary Figure S14).

### 2.10 Metabolic Flux Analysis

For the assessment of mitochondrial function, a Cell Mito Stress Test Kit (Agilent) was used to assay the oxygen consumption rate (OCR) on a Seahorse XFp Extracellular Flux Analyzer (Agilent). S + O + Z-transduced ADSCs and neural stem cell (NSC)-OPCs were seeded at 20,000 cells/well in poly-L-ornithine + laminin-coated Seahorse XFp Cell Culture Miniplates (Agilent) and maintained for 24 h in proliferative growth medium (NBB27 medium supplemented with EGF + bFGF + PDGF-AA; for converted cells, doxycycline was also added).

On the day of the experiment, the medium was changed to Seahorse XF DMEM, pH 7.4 supplemented with 1 mM pyruvate, 25 mM glucose, and 4 mM L-glutamine, and the plates were incubated for 1 h at 37°C without CO_2_. The assay is based on optical sensors that measure the oxygen concentration after the sequential addition of drugs that modulate cellular respiration (1 µM oligomycin, which blocks ATP synthase; 2 µM FCCP, a protonophore that enables maximum electron flux through the electron transport chain; and rotenone and antimycin A [RAA], which inhibit complexes I and III, respectively, thus shutting down mitochondrial activity), allowing to estimate the basal, ATP-linked, or maximal capacity of mitochondrial respiration.

### 2.11 Myelination in Culture

Dorsal root ganglion neurons (DRGns) were obtained from the cervical enlargement of E16 rat embryos (E0 being the day after overnight insemination) and dispersed using 0.05% trypsin + 0.02% EDTA digestion for 15 min at 37°C on a shaker, followed by mechanical trituration with a P1000 automatic pipette fitted with a fire-smoothened sterile filter tip. The cell suspension was passed through a 70-µm mesh cell strainer to eliminate undigested tissue (containing blood vessels, meninges, and nerve fragments) and the cells were then seeded on poly-L-ornithine + laminin-coated coverslips (diameter: 12 mm, placed inside 24-well plates) at approximately 1,000 cells/coverslip in DMEM supplemented with 10% FBS and 50 ng/ml β-NGF overnight. The next day, the cultures were changed to NBB27 medium containing β-NGF + 10 µM fluorodeoxyuridine + 10 µM uridine for 2**–**3 days to eliminate dividing cells (comprising mostly Schwann cells and perineurial fibroblasts). The medium was subsequently replaced with NBB27 medium supplemented only with β-NGF for 2**–**3 days. The cycle (with/without antimitotics) was repeated twice more. After 2 weeks, the culture consisted of small clusters of neurons interconnected by a mesh of axonal fascicles free of Schwann cells. A 20 µL drop of S + O + Z-transduced ADSCs was carefully deposited at the center of the neuronal culture. After a few minutes, these cells had dispersed and adhered to the axons and the coverslip. Myelination was allowed to progress for 3**–**7 more weeks in NBB27 medium containing 0.5 μg/ml doxycycline. For some experiments, 1.5 µM benztropine mesylate was added to co-cultures for the last 1**–**2 weeks.

### 2.12 Transmission Electron Microscopy

Transmission electron microscopy (TEM) was performed using various procedures, the most consistent one of which is described here. A 20-mm^2^ area of a 35-mm Petri dish was coated with rat tail collagen type I (4.36 mg/ml in 0.02 N acetic acid, spread with cell scrapers), polymerized under NH_4_OH vapor for 2 min, and allowed to dry. Straight tracks (12 × 1.5 mm) were drawn on the collagen with Geltrex (LDEV-Free Reduced Growth Factor Basement Membrane Matrix, from Gibco) or laminin (each at 4 µL per track), and allowed to dry. The Petri dish was rinsed once with HBSS, filled with 1 ml of DMEM supplemented with 10% FBS (D10 medium) + 100 ng/ml β-NGF, and kept in an incubator until DRG seeding. For DRG seeding, 400 µL of medium was removed from the plate, leaving only a thin layer of liquid, and two DRG were positioned at each end of the tracks. The ganglia were induced to attach to the substrate by incubating overnight inside a humidified chamber, after which the medium was switched to NBB27 medium supplemented with β-NGF (50 ng/ml) + fluorodeoxyuridine (10 µM) + uridine (10 µM), as described above. After three cycles of antimitotic treatment, the DRGns had extended numerous straight axons along the tracks, merging with those extending from the opposite ganglions, such that a cable of nude axons had formed. Then, the medium was changed to NBB27 medium + doxycycline, and 4 µL of a concentrated suspension (20,000 cells/µL) of S + O + Z-transduced ADSCs were carefully deposited along each axonal cable under a microscope (after 10 min, numerous oligodendroglia-like cells had attached to axons; see Supplementary Figure S15).

After at least 4 weeks of co-culturing, the cells were fixed for 5 min by adding to the medium an equal volume of 4% paraformaldehyde in PBS, and then maintaining them in 2% paraformaldehyde + 2.5% glutaraldehyde in PBS at 4°C until processing for TEM. Post-fixing/staining in 1% OsO_4_, dehydration in alcohol, and embedding in epoxy resin were performed in the Petri dish, leaving a thin layer of epoxy resin covering the cells that was allowed to harden overnight at 56°C (as shown in Supplementary Figure S15E). Fragments of the central portion of the axonal cable were cropped and positioned in resin molds for transverse cutting in an ultramicrotome (Leica Ultracut S). Ultrathin (60 nm) sections were contrasted on grids with uranyl acetate and lead citrate and examined in a Jeol JEM1010 (100 kV) microscope at the TEM facilities of the School of Medicine of the Autonomous University of Madrid or a Jeol JEM1400 Flash at the Electron Microscopy Service of the Centro de Biología Molecular “Severo Ochoa” (CSIC/UAM, Madrid).

## 3 Results

### 3.1 Immature Oligodendroglia Can Be Efficiently Generated by Expressing *Sox10*, *Olig2*, and *Zfp536* Transgenes in Adult Rat Adipose Tissue-Derived MSCs

**FIGURE 1:**
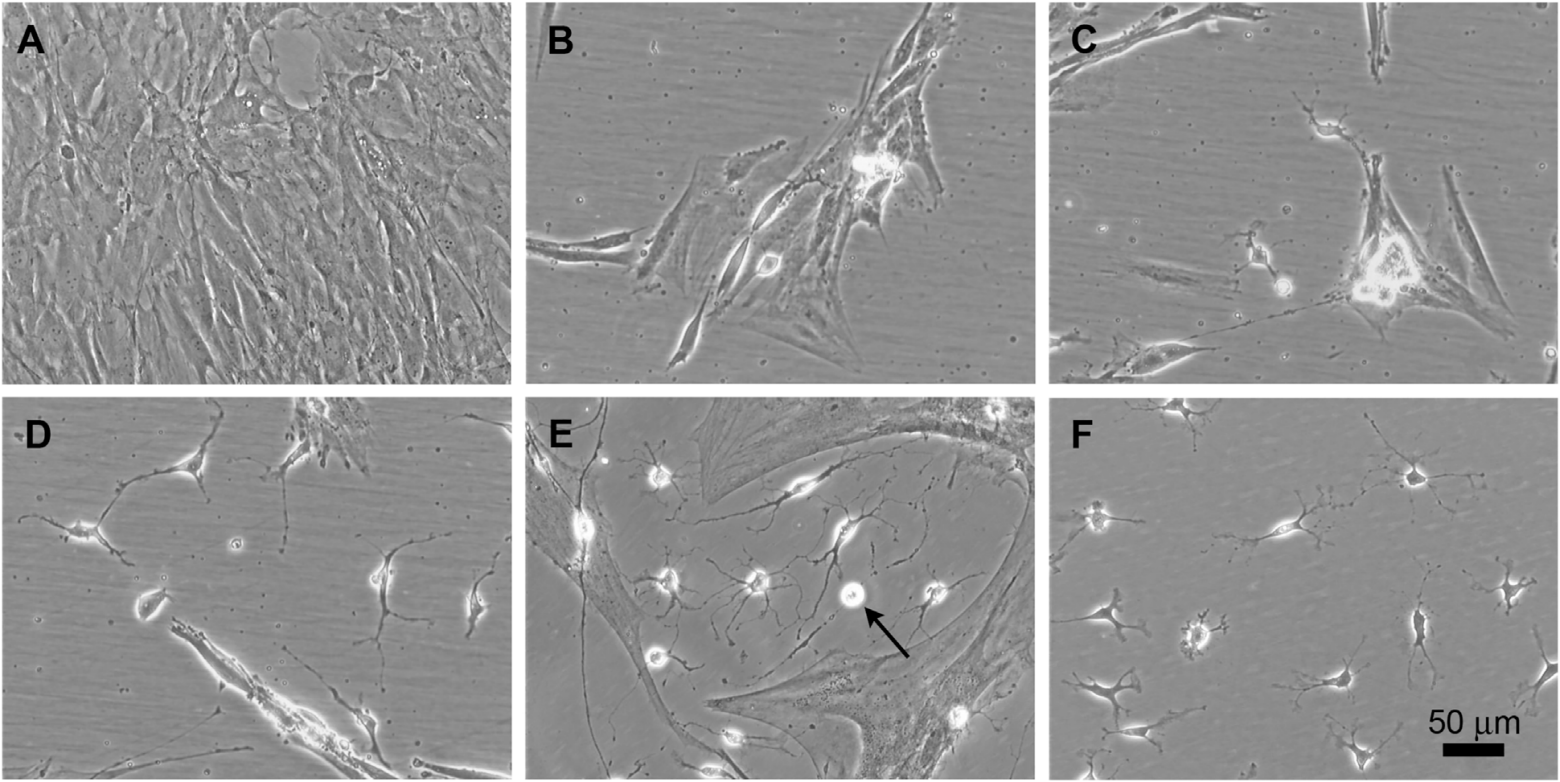
Time course of phenotypic conversion from adult rat ADSCs into oligodendroglia-like cells. Phase contrast images at different stages after the activation of the transgenes. **(A)** Confluent culture of ADSCs in α20. **(B)** During the first week of transgene activation, some refringent cells are visible between groups of flat cells. **(C)** Refringent cells migrate to empty areas of the culture surface but keep contact with clusters of flat cells. **(D)** By 3–4 weeks, independent branched and refringent cells are observed. **(E)** By 6 weeks after transgene induction, colonies of small and ramified cells are prominent. These cells proliferate in the culture conditions here provided (arrow points to a cell undergoing mitosis). **(F)** At 3 months, a high proportion of cells in the culture show small and refringent cell bodies with a few branches.

**FIGURE 2:**
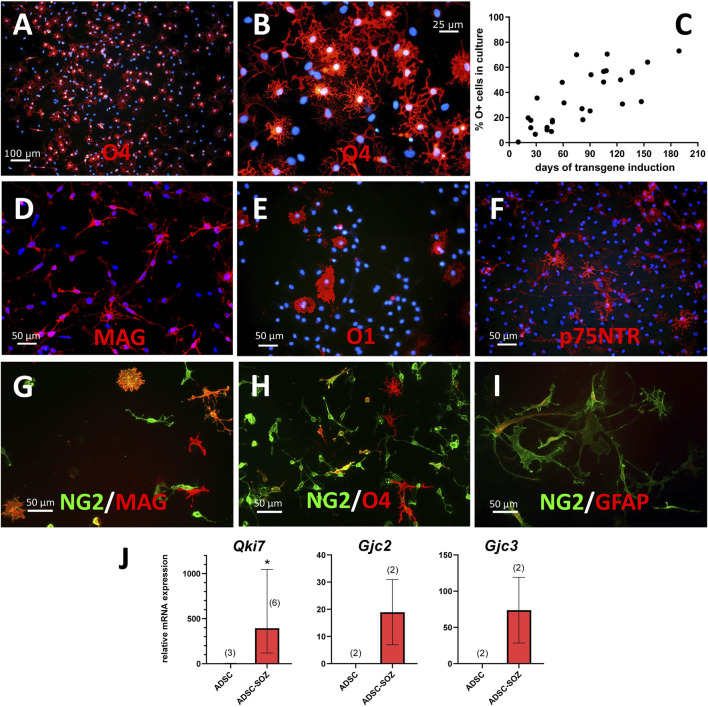
Phenotypical characterization of cells generated by transduction of Sox10 + Olig2 + Zfp536. **(A, B)** After 3 months of continuous expansion, around 50% of cells in the culture are O4^+^ (shown in red at two different magnifications; in blue, bisBenzimide Hoechst 33342- counterstain of nuclei). **(C)** Percentage of O4^+^ cells in cultures of converted cells, examined at different times of S + O + Z transgene expression. **(D)** MAG is the earliest oligodendroglial-specific marker of cell conversion that we have observed, shown here in a 2-months culture. **(E)** Under continuous induction of transgene expression by doxycycline in proliferating cultures, a few cells show O1 staining with limited branching. **(F)** Multiple cells show p75NTR expression at their surface. **(G–I)** NG2^+^ cells (green) in double labelling (in red) with MAG **(G)**, O4 **(H)** or GFAP **(I)** after 2-months of transgene activation. Double-labelled cells show orange color. Notice that, besides many double-labelled cells, there are singly-labelled cells for MAG or O4, as well as cells that are labelled for NG2 only. Light expression of GFAP can be observed in some NG2^+^ cells **(I)**. Scale bars, indicated in each picture. **(J)** Demonstration of *Qki7* and oligodendroglial-specific connexins Cx47 (gene *Gjc2*) and Cx29 (gene *Gjc3*) mRNA expression in S + O + Z-transduced ADSCs (ADSC-SOZ), while control rat ADSC in basal conditions show no or very low expression. Columns represent medians ± range, with the sample number (each with technical triplicates) indicated between parentheses. For *Qki7*, Mann-Whitney U test, shows statistically significant differences (*p* < 0.05) to control ADSC.

ADSCs were transduced with tetracycline-inducible *Sox10*, *Olig2*, *Zfp536*, and/or *Nkx6.1* (N) transgenes to test if the exogenous expression of the encoded transcription factors could induce the conversion of these adult rat cells into oligodendroglia. The expression of *Sox10*, *Olig2*, and *Zfp536* in a defined medium containing EGF (20 ng/ml) + bFGF (20 ng/ml) + PDGF-AA (10 ng/ml) led to the generation of colonies containing a few small, branched, and refringent cells within 3**–**4 weeks (Figure 1). Immunofluorescence staining of these cultures showed the presence of O4^+^ cells (Figures 2A,B), which we considered to be presumptive OPCs as this marker has not been previously observed in ADSCs (Vellosillo et al., 2017). Although morphological changes could be observed in these branched and refringent cells from the first week (Figure 1B), it was not until they decreased their soma size, divided, and became separated from the flat, mesenchymal-like cells that they could be labeled by the O4 antibody. These oligodendroglia-like cells proliferated and formed larger colonies during the following weeks, while the rest of the cells in the culture either died or proliferated slowly. The proliferation of these oligodendroglia-like cells accelerated from month two of the induction of *Sox10* + *Olig2* + *Zfp536* transgene expression, when there was a doubling of the cell population every 3.9 days, to month three and subsequently, when there was a doubling every 1.8 days, such that culture maintenance required weekly detachment of cells with accutase and the seeding in a new flask at a 1:15 dilution. By month 3, O4^+^ cells constituted approximately 40% of the total cell population (Figures 2A–C; Supplementary Figure S1). Cells in these cultures could continue proliferating for longer than 7 months, whereas the original ADSCs typically became senescent by 4–5 weeks). At these times, the proportion of O4^+^ cells in the culture was >70%, without any type of selection.

Myelin-associated glycoprotein (MAG) was also expressed in the small, refringent, and branched cells from very early after the induction of *Sox10* + *Olig2* + *Zfp536* transgene expression (Figure 2D). MAG could also be considered an oligodendroglial-specific marker as its expression was not observed in mesenchymal cells (Vellosillo et al., 2017). Indeed, MAG positivity could be considered the most reliable marker of ADSC conversion into oligodendroglia-like cells (see below). Additionally, MAG^+^ cells could constitute >90% of the total number of cells after 3 months or longer of transgene expression.

The S + O + Z-transduced cultures also contained minor populations of poorly-developed O1^+^ cells (Figure 2E). This labelling was lost from ADSCs after 3 weeks in culture conditions favoring OPC growth, but could be detected in refringent, branched cells at approximately 14 weeks. This O1^+^ population never accounted for more than 20% of cells when the culture was maintained in proliferation medium (statistics from an example culture are shown in Supplementary Figure S1).

Some S + O + Z-transduced cells also expressed GFAP, which is a characteristic of astrocytes. GFAP immunofluorescence was observed to label cytoskeletal filaments in both protoplasmic-like and filamentous-like cells (Figure 2I).

A small population of cells showing membrane expression of the low-affinity NGF receptor (p75NTR) appeared by 2 months of transgene expression (Figure 2F; Supplementary Figure S1). These p75NTR^+^ cells displayed an elongated and branched morphology, to some extent distinguishable from that of O4^+^ cells, and also different from that of typical Schwann cells in culture.

No membrane labelling with A2B5 antibody was observed in any of our transduced cultures. In contrast, staining with this antibody was strong in neural OPC cultures (see Supplementary Figure S14D). The repeated absence of A2B5 labelling in S + O + Z-transduced cells suggested that oligodendroglia and astroglia-like cells were not generated through the so-called O2A stage.

Unlike A2B5, numerous NG2^+^ cells were present in S + O + Z-transduced ADSC cultures (Figures 2G–I) from the earliest stages. However, NG2 also labeled untransduced ADSCs, and thus could not serve as a marker of oligodendroglial conversion. Indeed, at shorter induction times (i.e., <2 months of *Sox10* + *Olig2* + *Zfp536* transgene expression) only a relatively small population of NG2^+^ cells was co-labeled with the O4 antibody (Figure 2H). Similarly, only a sub-population of NG2^+^ cells co-expressed MAG (Figure 2G). Some ADSCs that had not been transduced and expressed smooth muscle actin (SMA) were also NG2^+^ (not shown). These observations suggested that NG2 expression was not associated with oligodendroglial conversion in S + O + Z-transduced ADSCs.

Cultures of converted cells also expressed QKI-7, an isoform of rat quaking, an RNA-binding protein that is highly upregulated in myelinating oligodendrocytes in the CNS and is labeled with the monoclonal antibody CC1 (Bin et al., 2016). RT-qPCR analysis showed that although *Qki7* mRNA was barely detectable in ADSCs maintained in NBB27 medium supplemented with EGF + bFGF + PDGF-AA, consistent *Qki7* expression could be detected in S + O + Z-transduced cells maintained in the same medium and under the same conditions (Figure 2J; see also Supplementary Figure S7A).

Additionally, RT-qPCR analysis also showed the presence of mRNAs for oligodendroglial-specific connexins in converted cells (Figure 2J). Both *Gjc2* (encoding Cx47) and *Gjc3* (encoding Cx29) were expressed in S + O + Z-transduced cells, but not in untransduced ADSCs, when maintained in the same medium (NBB27 medium supplemented with EGF + bFGF + PDGF-AA) and under the same conditions.

### 3.2 Differentiation of S + O + Z-Transduced Cells

**FIGURE 3:**
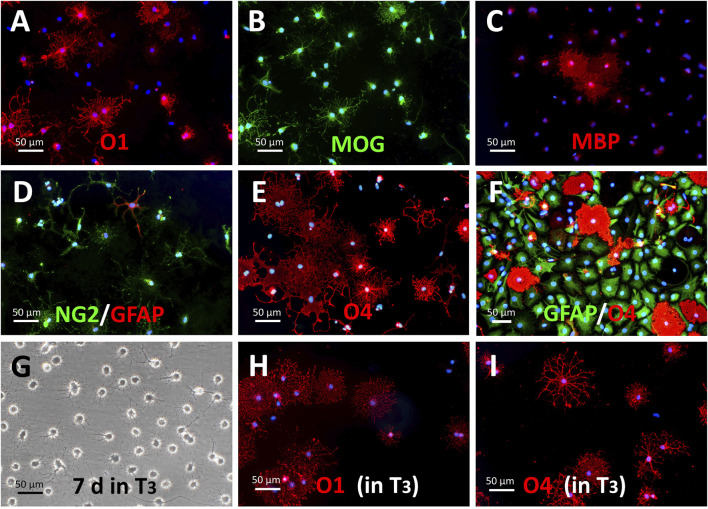
Maturation of S + O + Z-transduced cells in oligodendroglial-differentiating conditions. **(A–E)** Withdrawal of growth factors from the culture medium (i.e., switching to NBB227 + doxycycline) initiates the differentiation of OPC-like into mature oligodendrocyte-like cells. **(A)** Numerous O1^+^ cells with extended cytoplasms after 10 days of growth factor withdrawal are shown. **(B)** MOG^+^ cells (same field as in **(A)**). **(C)** MBP^+^ cells. **(D)** NG2^+^ cells increase morphological complexity but there is not further increase in size or number of GFAP^+^ cells. **(E)** O4 antigen keeps its expression in these conditions but most cells show profuse morphologies. **(F)** In the presence of DRG neurons and kept in NBB27 + doxycycline for 6 days, GFAP^+^ (in green) of the protoplasmic type and well-developed O4^+^ cells (in red) make up most of the culture cell population. **(G–I)** Supplementation of NBB27—doxycycline medium with T_3_ further enhances morphological differentiation. **(G)** Phase contrast image of living 5 month-old S + O + Z-transduced ADSC culture maintained for the last 7 days in NBB27 + T_3_ + doxycycline. The overall morphology is homogeneous and resembles pure oligodendrocyte cultures. Sibling cultures of the cells in G, immunostained for O1 **(H)** or for O4 **(I)**, show enhanced differentiation, taking shorter times to reach larger and more profuse branching.

The withdrawal of growth factors from the culture medium elicited the appearance of large numbers of O1^+^ (from approximately 1%–28.35 ± 4.35% in quantification of four cultures) and MBP^+^ cells with more mature morphologies (i.e., larger and more complex arborizations). Addition of T_3_ (60 nM) to the medium further stimulated the O1^+^ cell abundance and morphological complexity (Figures 3G,H). It should be considered, however, that the qualitative formula of B27 supplement includes T_3_ at an undisclosed concentration (Brewer et al., 1993). O4 antigen expression was maintained when cells were differentiated through growth factor withdrawal or T_3_ stimulation (Figures 3E,I). The withdrawal of EGF and bFGF, but not PDGF-AA from the medium could also enhance the maturity of converted cells. Under this condition, the expression of PDGF receptor alpha gene (*Pdgfra*) was reduced (Supplementary Figure S7A), implying that the cells behaved as if they had been maintained in growth factor-free NBB27 medium.

**FIGURE 4:**
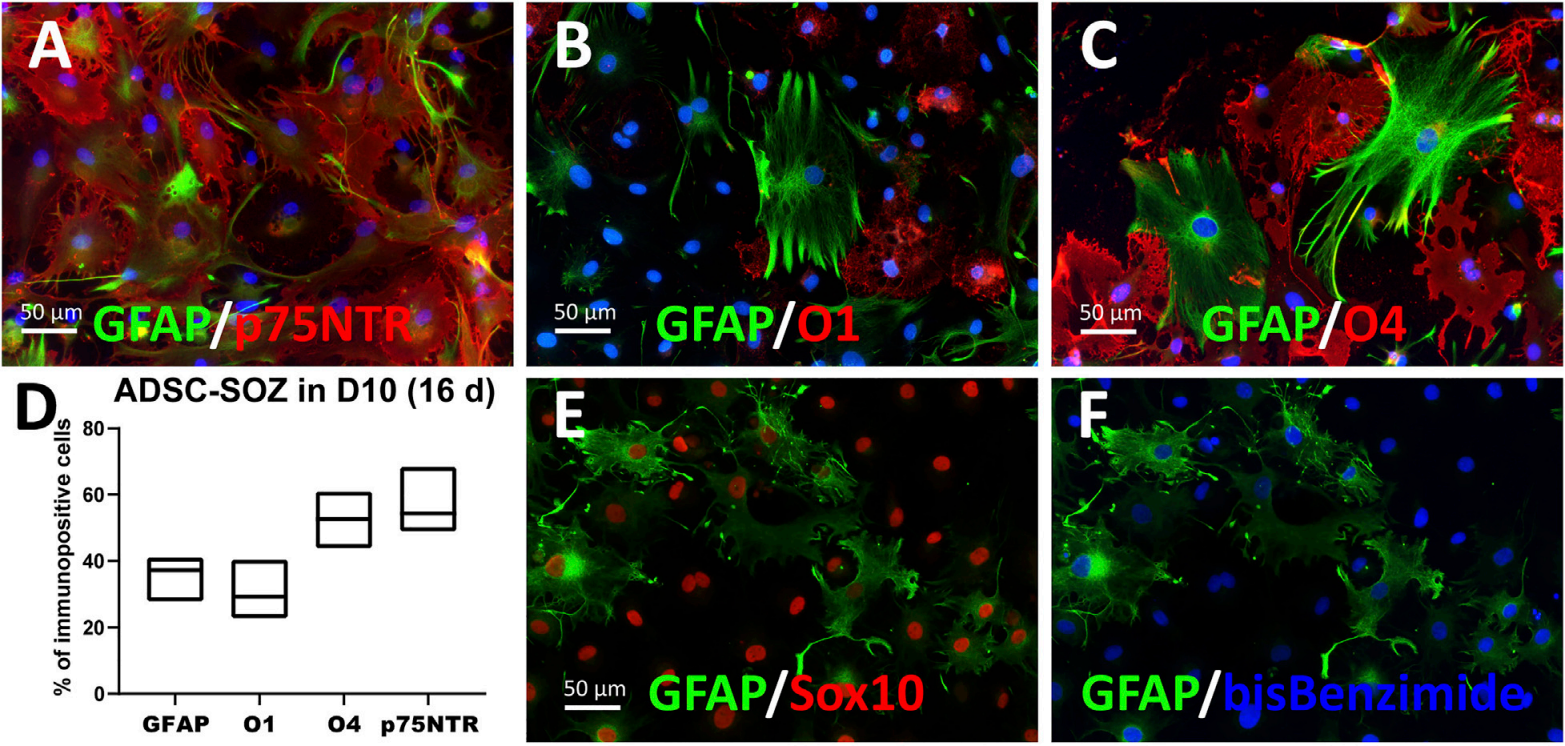
Differentiation to various glial phenotypes by culturing in 10% FBS-containing medium. **(A–D)** ADSCs expressing S + O + Z for 3 months and cultured for the last 16 days in 10%-supplemented medium (D10 + doxycyline) change their morphology and cell composition. In **(A)**, many GFAP^+^ cells, of typical astrocyte-like morphology, are double-labelled as p75NTR^+^ cells. **(B)** O1^+^ cells showed a flat, non-branching morphology in D10 and no O1^+^ cell co-stained for GFAP. **(C)** By contrast, part of O4^+^ cells showed more elaborated morphologies and approximately 20% were co-stained for GFAP. In **(D)** graphical representation of % cells that were immunofluorescently labelled for each marker (means and range, n = 4–6 cultures). **(E, F)** ADSCs expressing S + O + Z for 5 months but maintained for the last 18 days in D10 + doxycycline medium. Sox10^+^ nuclei (in red immunofluorescence) made up 98% of all cell nuclei (stained with bis-benzimide, in blue) at this time of transgene activation. In **(E)**, it is shown that most (if not all) GFAP^+^ cells express the Sox10 transgene.

Protoplasmic GFAP^+^ cells, presumably astroglia, could be preferentially generated by switching the defined medium to D10 medium + doxycycline (Figure 4). Intensely-labeled GFAP^+^ cells constituted a significant proportion of the total cell population (averaging 35.86% of cells in 3**–**4 months cultures; range from 27.9% to 41.05%). In these differentiating conditions, S + O + Z-transduced cells exhibited reduced proliferation as well as enlarged nuclei and cytoplasm. Seven days after switching to D10 medium containing doxycycline, most of the cells displayed a flat morphology and expressed p75NTR (56.3%) and/or O4 (51.4%) in the same proportions as observed in proliferation medium (see also Supplementary Figure S11). Although double immunostaining for p75NTR and O4 could not be performed, since both single-labeled cells exceeded the 50% it was evident that, at least, a subpopulation of the cells expressed both markers. Similarly, many NG2^+^ cells were co-labeled with GFAP. Interestingly, after 1 month or longer in D10 medium, an O1^+^ sub-population of cells displayed a flat morphology with multiple peripheral processes, and did not co-express GFAP. Also interestingly, nearly all GFAP^+^ cells showed nuclear Sox10 expression (due to transgene expression) (Figures 4E,F); additionally, large numbers of intensely stained GFAP^+^ cells were observed in NBB27 medium when S + O + Z-transduced cells were co-cultured with DRGns (Figure 3F).

### 3.3 Molecular Characteristics of Converted Cells

**FIGURE 5:**
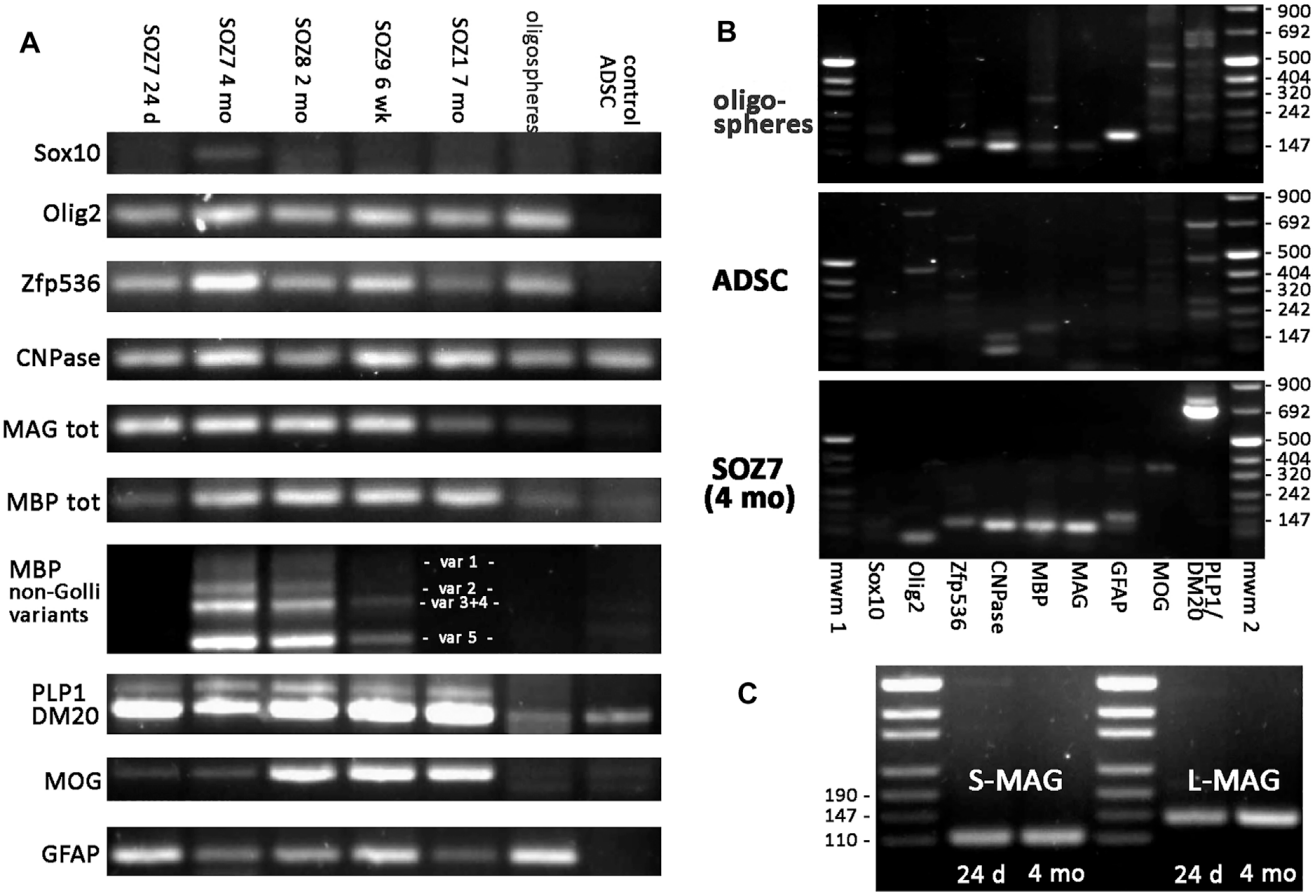
End-point RT-PCR of S + O + Z-transduced adult rat ADSCs. Expression of endogenous mRNAs of *Sox10*, *Olig2* and *Zfp536* transcription factors, as well as those of myelin-related proteins (CNPase, MAG, MBP, PLP1/DM20, and MOG), and of astroglial protein GFAP, in different samples. SOZ1, SOZ7, SOZ8 and SOZ9 correspond to S + O + Z-transduced ADSCs from different animals at different times of transgene activation, ranging between 24 days and 7 months. Additionally, mRNA expression in proliferating neural oligospheres and in untransduced adipose-derived MSC (control ADSC) is shown for comparison. Amplicon sizes are detailed in Supplementary Table S3. **(A)** Comparative RT-PCR of the different glial molecules. Notice that endogenous *Sox10* is barely expressed. Endogenous *Olig2* and *Zfp536* mRNAs are not detected in control ADSC, but they are expressed in S + O + Z-transduced cells, from very early, as well as in oligospheres. CNPase is expressed in all samples, including control ADSC. MAG is also expressed in converted cells from early times, is very low in oligospheres, and totally absent in ADSC. Expression of the various isoforms of myelin-related proteins is shown for PLP1/DM20 (PLP1 and DM20 isoforms), MBP (non-Golli variants 1–5) and MOG (with or without signal peptide). **(B)** Comparison of the set of RT-PCRs assayed in oligospheres, control ADSCs and 4 months-activated S + O + Z-transduced cells. mwm1, mwm2: DNA molecular weight markers. **(C)** Expression of the two isoforms of MAG (S-MAG and L-MAG) at two different times of transgene activation (24 days and 4 months) showing that both isoforms are neatly expressed at early times. All PCRs were performed for 35 cycles using the same starting amount of cDNA.

End-point RT-PCR analyses at 24 days, 6 weeks, 2, 4, and 7 months of continuous *Sox10*, *Olig2*, and *Zfp536* transgene expression in ADSCs identified the presence of mRNAs for the expected isoforms of key proteins involved in myelination. For comparison, untransduced ADSCs and oligospheres derived from neural tissues were simultaneously analyzed. As shown in Figure 5, *Cnp* (encoding CNPase), *Mbp*, *Mog* (isoforms containing or not the signal peptide), *Mag*, and *Plp1/Dm20* were expressed in S + O + Z-transduced cultures. As expected, untransduced ADSCs expressed *Cnp*, the *Dm20* variant of the *Plp1* gene, and low levels of *Mbp* and *Mog* (Vellosillo et al., 2017). Oligospheres, which showed little spontaneous glial differentiation, also expressed *Cnp* and low levels of *Mbp*, *Mag*, *Mog*, and *Plp1/Dm20*. Notably, S + O + Z-transduced ADSCs mainly expressed *Mbp* variant 5 (the shortest) as well as the rest of non-Golli variants to varying degrees, showing more intense bands with continued transgene activation. Untransduced ADSCs displayed very weak expression of some *Mbp* variants, while oligospheres did not show differentiated bands of *Mbp* variants.

A well-defined *Gfap* band was shown in S + O + Z-transduced ADSCs from 24 days of transgene activation as well as in oligospheres, but not in untransduced, control ADSCs.

The endogenous expression of *Sox10*, *Olig2*, and *Zfp536* was also evaluated using primers directed at the 5′ untranslated region of their mRNAs (including an intron), as the transgenes contained only the coding sequences and would not be amplified using these primers. Consistent expression of endogenous rat *Olig2* and *Zfp536* was observed in S + O + Z-transduced cells; however, the expression of endogenous *Sox10* was close to the limit of detection after 35 PCR cycles. Oligospheres expressed the genes coding for all three transcription factors. In contrast, no *Sox10*, *Olig2*, or *Zfp536* expression was detected in untransduced ADSCs maintained in OPC medium (Figures 5A,B).

**FIGURE 6:**
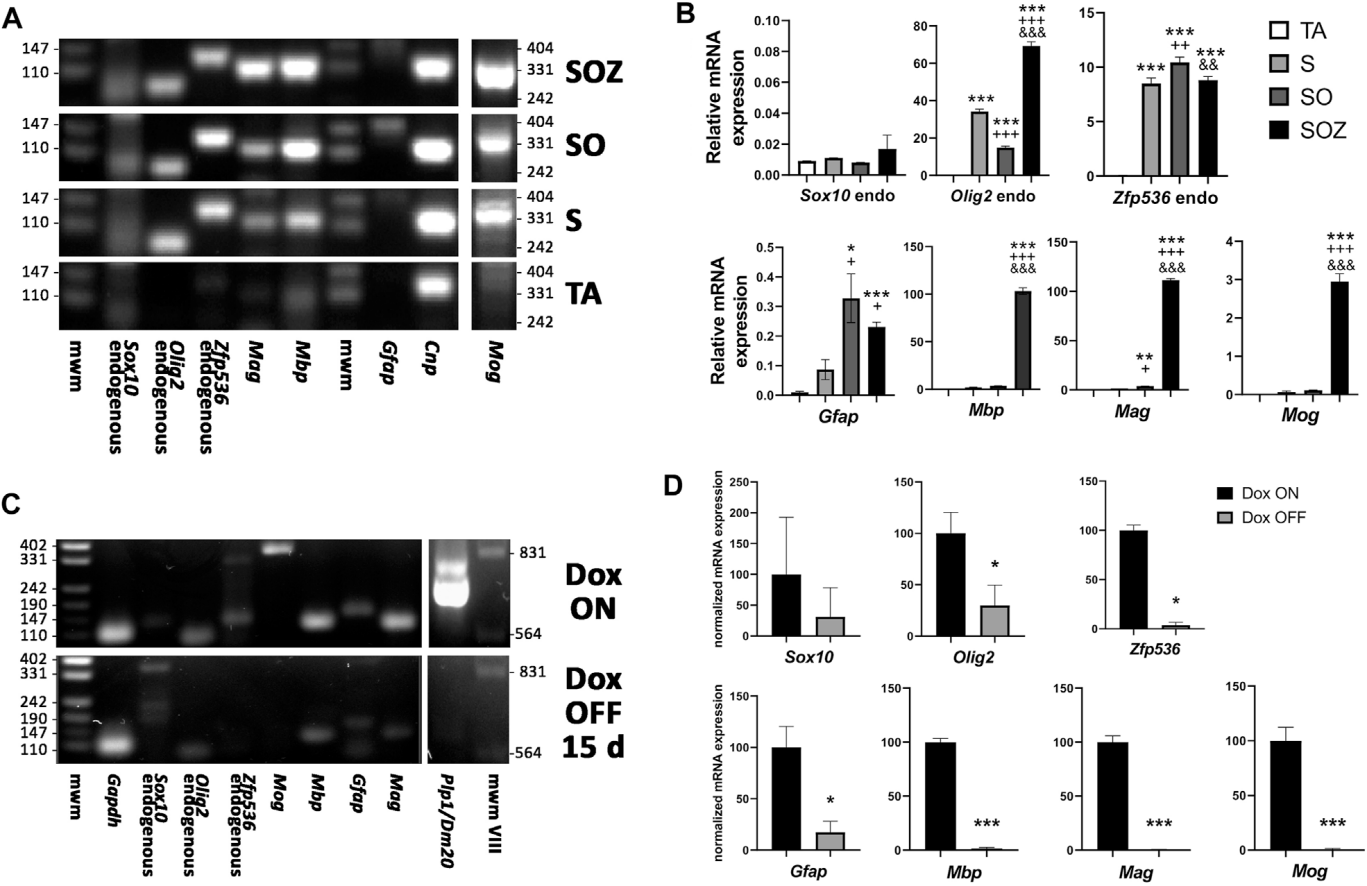
Effect of transgene activation on mRNA expression of endogenous transcription factors and glial genes. **(A)** end-point RT-PCR of control rat ADSCs, transduced only with the transactivator (TA) and of ADSCs additionally transduced with *Sox10* (S), *Sox10* + *Olig2* (SO) or *Sox10* + *Olig2* + *Zfp536* (SOZ). **(B)** real time RT-qPCR in the same conditions. Endogenous *Sox10* expression remains practically invariable in all these conditions, while *Olig2* and *Zfp536* mRNA expression is induced by S transduction, with additional changes in SO or SOZ transgene combinations. Expression of the myelin-related proteins (*Mbp, Mag, Mog*) is strongly induced by the SOZ transgene combination. In SO combination, only *Mag* shows statistically significant increase, although at low level. By contrast, *Gfap* mRNA is induced by S, SO or SOZ transgene expression. Except for *Sox10*, all gene statistics show ANOVA significant differences (*p* < 0.001). Post-hoc Tukey’s multiple comparison tests were performed; n = 3. Symbols: *, statistical significant differences with respect to TA; +, with respect to S; $, with respect to SO. One symbol, *p* < 0.05; two symbols, *p* < 0.01; three symbols, *p* < 0.001. **(C)** end-point RT-PCR and **(D)** real-time RT-qPCR, after 6 months of S + O + Z expression followed by doxycycline maintenance (Dox ON) or withdrawal (Dox OFF) from the culture medium for 15 additional days. Turning-off transgene expression in Dox OFF cultures greatly diminished or even abolished the expression of endogenous *Sox10*, *Olig2* and *Zfp536* mRNAs, as well as those of *Mbp*, *Mag*, *Mog*, *Plp1* and *Gfap*. n = 3. Data shown in graphics are normalized to each respective Dox ON sample average and analyzed by paired data *t* test. *p < 0.05; **p < 0.01; ***p < 0.001. mwm: DNA molecular weight marker.

Other transcription factor combinations were less efficient at inducing the generation of oligodendroglia-like cells from adult rat ADSCs. As shown by RT-qPCR, *Sox10* transgene expression alone could induce the endogenous expression of *Olig2* and *Zfp536*, whereas endogenous *Sox10* expression was barely detectable. The mRNA expression of *Mag*, *Mbp,* and *Mog* was also induced by exogenous *Sox10*, while GFAP was expressed at low levels. The co-expression of the *Sox10* and *Olig2* transgenes further increased the expression levels of myelin proteins. Expression of the full set of transgenes (*Sox10*, *Olig2*, and *Zfp536*) intensified *Mbp*, *Mag*, and *Mog* expression. No transgene was observed to stimulate the expression of its corresponding endogenous mRNA (Figures 6A,B).

On the other hand, cells expressing the *Sox10* transgene, but not those transduced with the M2rtTA (expressing the transactivator) alone, could proliferate when cultured in NBB27 medium supplemented with EGF + bFGF + PDGF-AA and were positive for O4 immunostaining. However, these cells displayed a flat, unbranched, and vacuolated morphology, with only sporadic cells expressing MAG. This demonstrated that *Sox10* transgene expression was sufficient to induce both O4 positivity and a proliferative response to EGF, bFGF, and/or PDGF-AA, but not for efficiently generating myelination-capable cells. ADSC cultures transduced with both *Sox10* and *Olig2* contained cells with some branching and O4 staining; MAG expression was also detected sporadically, as above, and only these MAG^+^ cells had the morphological characteristics of oligodendroglia (Supplementary Figure S3). GFAP immunostaining was not observed under any of the above-mentioned conditions.

The exogenous expression of other transcription factor combinations either was inefficient at inducing the production of O4^+^ cells or led to the generation of cells devoid of oligodendroglial phenotypical characteristics. Combinations that did not include the *Sox10* transgene (*Olig2* + *Zfp536* or *Olig2* only) did not generate cells with oligodendrocyte morphology unless the transduced cells were treated with 1 mM RA for 4 days before transgene activation, in which case it took 6 weeks for small, refringent, branched cells to appear (see Supplementary Table S2).

The inclusion of the *Nkx6.1* transgene (encoding a transcription factor) in the transgene combination was unfavorable for the generation of O4^+^ cells from adult rat ADSCs. The S + O + N combination did not produce colonies of cells with oligodendroglial morphology (Supplementary Table S2); additionally, while the S + O + Z + N combination produced small, refringent, and branched cells, relatively few were O4^+^. However, pre-treating S + O + Z + N-transduced cultures with RA increased the number of O4^+^ cells, even though their morphology was, to some extent, aberrant, and also led to the generation of larger numbers of p75NTR^+^ cells (Supplementary Figure S5).

### 3.4 Continuous *Sox10*, *Olig2*, and *Zfp536* Transgene Expression Was Required for the Maintenance of the Oligodendroglial Phenotype

The continuous induction of transgene expression with doxycycline was required to maintain the oligodendroglial phenotype. In cultures expressing the *Sox10*, *Olig2*, and *Zfp536* transgene combination for less than 3 months, the withdrawal of doxycycline from the culture medium was accompanied by an increase in size in former oligodendroglia-like cells, as well as the frequent build-up of lipid droplets, which confirmed their reversion into adipocytes (Supplementary Figure S4). Oligodendroglia-like cells reappeared if the culture medium was again supplemented with doxycycline. Even after 6 months of continuous oligodendroglial-like growth, the withdrawal of doxycycline for 14 days arrested cell proliferation, induced a morphological change into flat and elongated cells, and led to cell apoptosis. This indicated that the cells were not responsive to the EGF, bFGF, and/or PDGF-AA growth factors still present in the medium, resulting in fewer cell numbers. Sibling cultures maintained with doxycycline continued to proliferate and showed oligodendroglial morphology. Analyses of mRNA expression in these long-term cultures confirmed that doxycycline withdrawal for 14 days was sufficient to lower the expression of endogenous *Olig2*, *Zfp536*, and *Gfap* to barely detectable levels, as well as abolish the expression of genes (*Plp1*, *Mog*, *Mag*, and *Mbp*) coding for myelin-related proteins (Figures 6C,D).

### 3.5 The Capacity of S + O + Z-Transduced Cells to Function as Oligodendroglia

Three different studies were performed to analyze the capacity of these converted cells to function like oligodendroglia.

#### 3.5.1 Evaluation of the Expression of Neurotransmitter Receptors With Roles in OPC Function

RT-qPCR was used to evaluate the expression of AMPA, NMDA, kainate, and dopamine receptors in S + O + Z-transduced ADSCs. These factors have been shown to participate in proliferative, developmental, and/or metabolic support of neural OPCs. We measured the levels of the AMPA R2, R3, and R4 subunits, which account for all AMPA receptors, as well as that of the NMDA-R1 subunit, which associates with all NMDA receptors. We also assessed the expression levels of the required subunits of the kainate receptor (GluK1, GluK2, and GluK3), as well as those of the non-essential but high-affinity-conferring subunits GluK4 and GluK5 (formerly KA1 and KA2, respectively). Finally, we sought to detect the presence of D1-type (DR1 and DR5) and D2-type (DR2, DR3, and DR4) dopamine receptors, which we had observed were expressed in OPCs and participated in their proliferative and differentiation processes (unpublished data).

**FIGURE 7:**
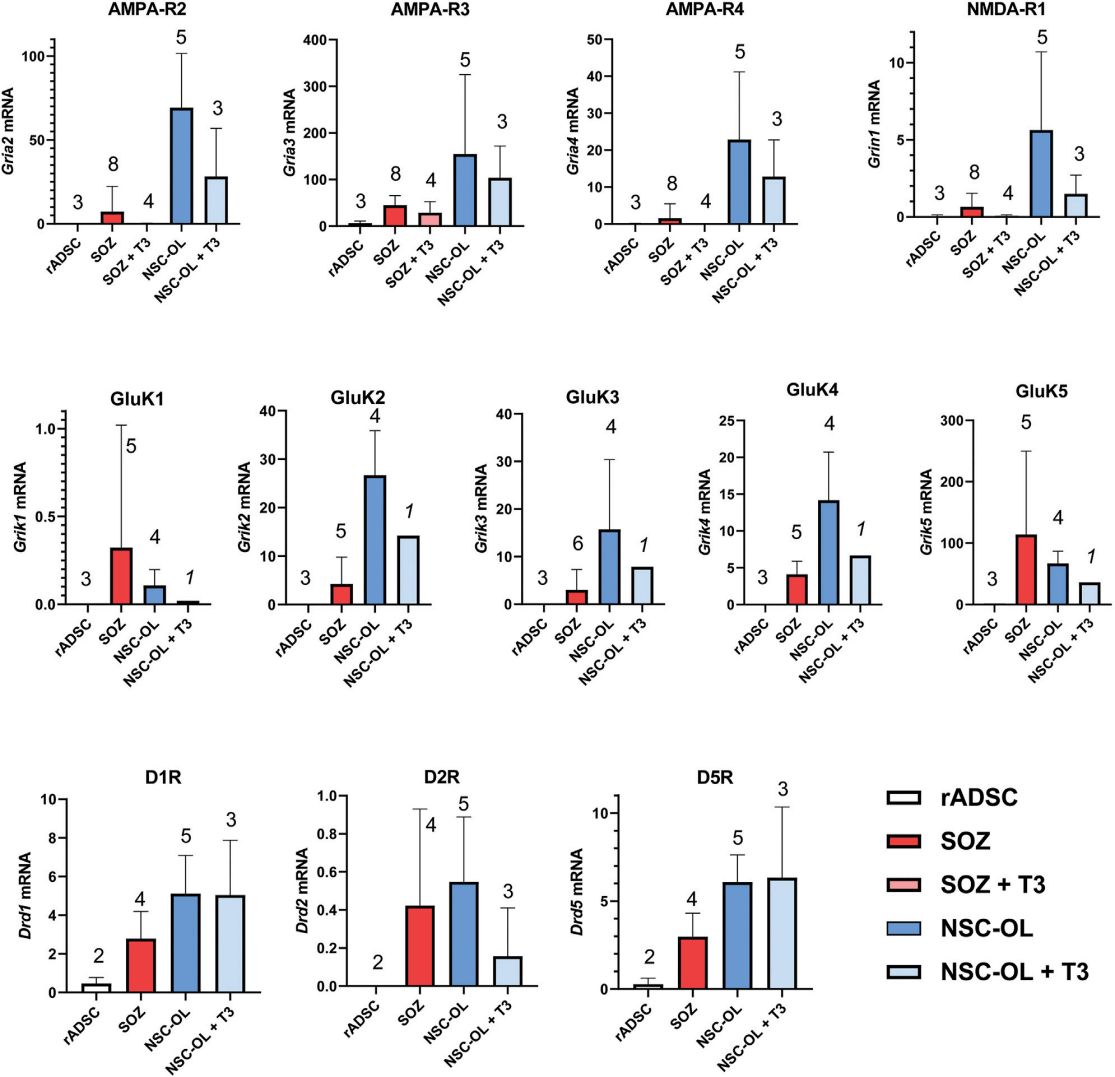
Expression of oligodendroglia-related neurotransmitter receptors mRNA by of S + O + Z-transduced ADSCs. Expression of all AMPA, kainate and NMDA receptor subunits present in the different glutamate receptors as well as DR1, DR2 and DR5 dopamine receptors were analyzed by RT-qPCR. Data represent the ratio of each gene cDNA relative to GAPDH cDNA multiplied by 1000. Numbers of assayed culture samples -each one with triplicate replicas-are shown on top of histogram columns. Our analysis demonstrates that S + O + Z-transduced ADSCs (SOZ) express many of these neurotransmitter receptors, some at low level and others, like the high-affinity GluK5 receptor, at high-level. By comparison, control, unconverted ADSCs showed null expression of most receptors and minimal expression, compared to SOZ, of AMPA-R3, GluK5 and D1-type receptors. Neural-derived oligodendroglia (NSC-OL) showed neat expression of all glutamatergic receptor subunits, except for GluK1, and low, but consistent, expression of DR1, DR2 and DR5 dopamine receptors. T_3_-treated cultures are also shown to check neurotransmitter receptor expression under oligodendroglial differentiating conditions. No statistical comparisons between all culture groups are provided due to low sample number in relation to data variability.

As expected, neural-derived OPCs in culture expressed all three AMPA receptor subunits as well as the NMDA-R1 subunit at various levels. Rat ADSCs maintained under basal conditions did not express mRNAs for these receptors, except for AMPA-R3 (although its mRNA levels were at the limit of detection using real-time PCR). In contrast, S + O + Z-transduced cells consistently expressed all these glutamate receptor subunits (Figure 7). Neural OPCs also expressed kainate receptors, a type of often disregarded glutamate receptor. In culture, neural OPCs expressed all five kainate receptor subunits at different levels, with GluK1 displaying the lowest levels of expression and GluK5 the highest. In untransduced ADSCs, the mRNA expression of the five kainate receptor subunits was undetectable. In contrast, the mRNA expression of all five subunits was detected in S + O + Z-transduced ADSCs (Figure 7).

The mRNA expression of the type 1 dopamine receptors (DR1 and DR5) was also detected in cultured neural OPCs and T_3_-differentiated oligodendrocytes. DR2, a type 2 dopamine receptor, was also expressed in neural OPCs, although its expression was downregulated in T_3_-differentiated cells. The expression of the DR3 and DR4 subtypes was not detected in neural oligodendroglia (not shown). Meanwhile, S + O + Z-transduced ADSCs consistently expressed DR1 and DR5 mRNAs; also, type-2 dopamine receptor mRNA was detected at low levels. Under basal culture conditions, untransduced rat ADSCs only showed very low mRNA expression levels of type 1 dopamine receptors (Figure 7).

Non-parametric Wilcoxon’s test was used to compare the expression of glutamatergic and dopaminergic receptors as a set, pairing the means of each receptor, in untransduced *vs*. S + O + Z-transduced adult rat ADSC (Supplementary Figure S13). This test demonstrated a very significant statistical difference (*p* < 0.0001) in the expression of the neurotransmitter receptor set of these two types of cells.

#### 3.5.2 Comparison of the Metabolic and Respiration Rates Between Converted Cells and OPCs

**FIGURE 8:**
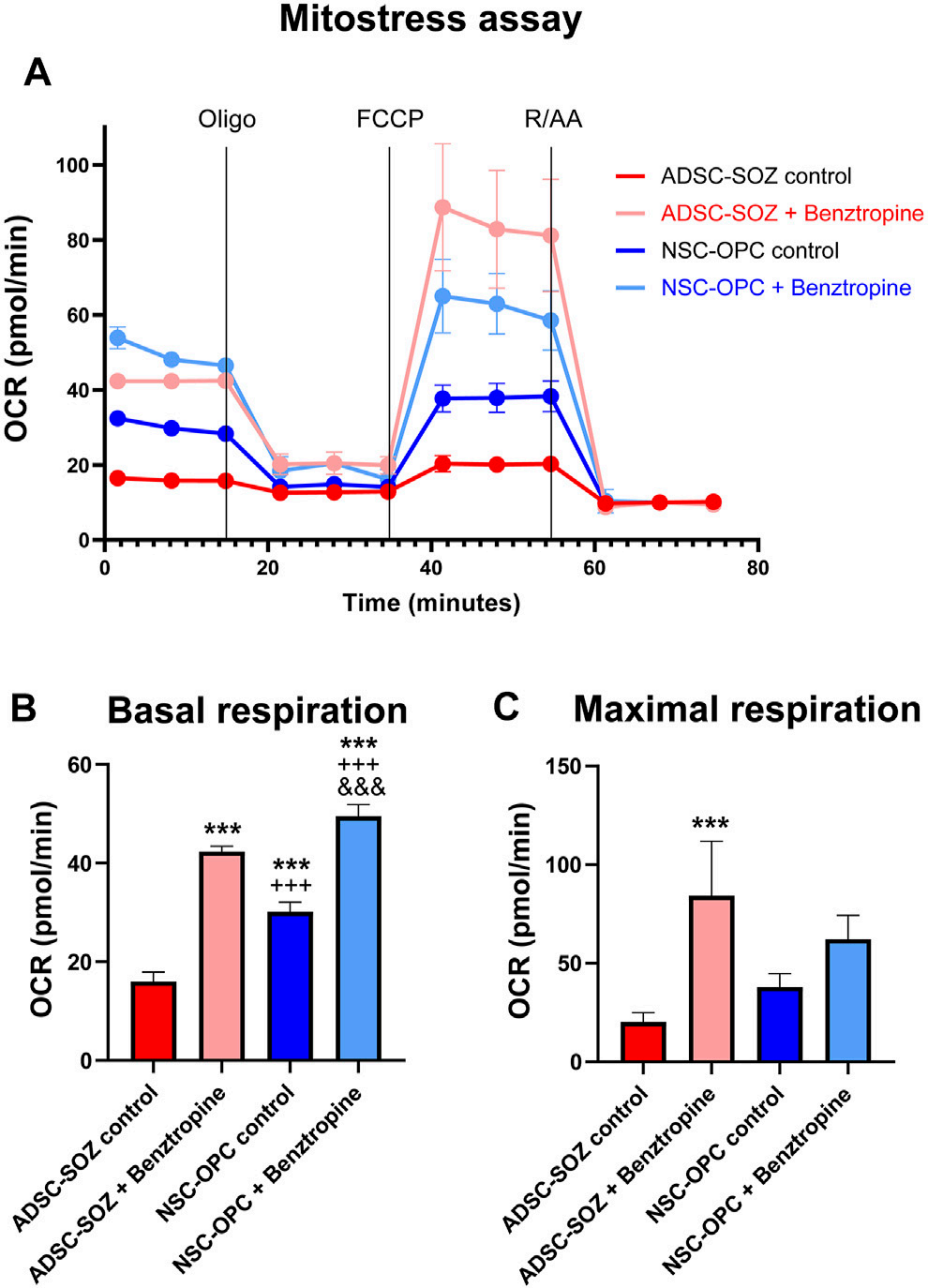
Analysis of mitochondrial function. **(A)** Mitochondrial function (Oxygen consumption rate, OCR) of S + O + Z-transduced ADSCs (ADSC-SOZ) and neural OPCs (NSC-OPC), in basal proliferative conditions or after treatment with benztropine (0.5 μM) for 6 days. OCR was measured at baseline, as well as after the successive addition of 1 μM oligomycin (Oligo), 2 μM FCCP and 2 μM rotenone and antimycin A (RAA), as indicated by vertical lines in the graphics. **(B)** Average measurement of basal respiration index. **(C)** Average of maximal respiration, from the above data. The differentiating effect of benztropine is accompanied by increases in cell respiration both in NSC-OPC and in ADSC-SOZ. Data show mean ± s.d. (n = 3–7 different samples). Symbols: *: statistical differences with respect to ADSC-SOZ; +: differences with respect to ADSC-SOZ with benztropine; &: differences with respect to NSC-OPC. One symbol, *p* < 0.05, two symbols, *p* < 0,01, three symbols, *p* < 0.001.

Cellular bioenergetics characterizes the differentiation state of oligodendroglia (Schoenfeld et al., 2010; Ziabreva et al., 2010). When OPCs differentiate, their mitochondrial metabolism rate is drastically increased. Benztropine is a repurposed drug that has been shown to promote oligodendrocyte differentiation (Deshmukh et al., 2013) and to increase the OCR (an index of mitochondrial functionality) in OPCs (Beyer et al., 2018). Using this oligodendroglia-specific action of benztropine, we tested whether it stimulated similarly S + O + Z-transduced ADSCs. NSC-OPC cultures showed a high rate of mitochondrial metabolism, in line with that previously reported (Sanchez-Abarca et al., 2001; Amaral et al., 2016), and S + O + Z-transduced ADSCs exhibited similar characteristics (Figure 8). Treatment with benztropine increased the basal respiratory capacity of NSC-OPCs and, to a greater extent, that of S + O + Z-transduced ADSCs (14.5 pmol/ml and 21.9 pmol/ml increases of their OCRs, respectively). In untransfected ADSCs, meanwhile, benztropine treatment led to a substantially lower OCR increase (2.7 pmol/ml, not shown in the graphic). The maximal respiratory capacity (in the presence of the ionophore FCCP) was also substantially increased in S + O + Z-transduced ADSCs and in NSC-OPCs (OCR: 47.9 pmol/ml and 11.1 pmol/ml, respectively). Therefore, when treated with benztropine, S + O + Z-transduced ADSCs show enhanced mitochondrial metabolic rates concomitantly with increased differentiation (see below), a property that is characteristic of neural oligodendroglia.

#### 3.5.3 The Myelinating Capacity of Converted Cells

Finally, we tested whether the converted cells could ensheathe and myelinate axons. Cultures of DRGns were obtained from E16 rat fetuses and purified by killing dividing fibroblasts and Schwann cells using antimitotic treatment. Cells from converted cultures, containing more than 50% O4^+^ cells, were seeded onto purified DRGn cultures and maintained in NBB27 medium supplemented with doxycycline for up to 7 weeks. Benztropine (1.5 µM) was added for the final 1**–**2 weeks to some co-cultures to test if this drug could promote myelination, as previously reported for OPCs (Deshmukh et al., 2013).

**FIGURE 9:**
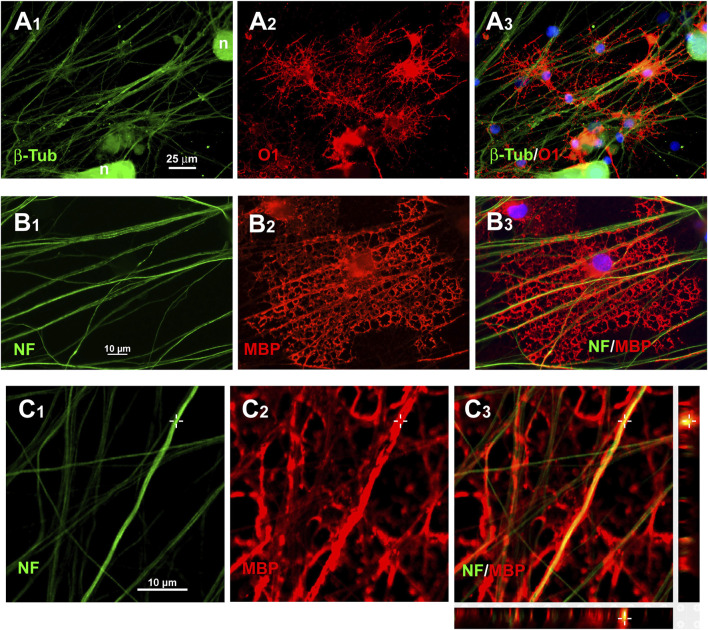
Immunostaining of axonal ensheathing by S + O + Z-transduced ADSCs. A_1_–A_3_: immunolabelling for β-tubulin isotype III (β-Tub) in green, to show axons, and O1/galactocerebroside, in red, to show oligodendrocyte surfaces, in co-cultures of DRG neurons (n) with S + O + Z-transduced cells. B_1_–B_3_: Immunolabelling for 200 kDa neurofilament, clone RT97 (NF, in green) and MBP (in red) showing an induced oligodendrocyte that forms large tubules of ensheathment along several axons. Nuclei are counterstained with bisBenzimide, in blue. C_1_–C_3_: confocal images of MBP^+^ processes around NF-labelled axons to show axonal ensheathment by converted cells; below and to the right of C_3_, the projections in the X-Z and Y-Z axes at the level indicated by the cross show that MBP labelling encircles NF-labelled axons.

Immunofluorescence staining for MBP or O1 showed that the processes of S + O + Z-transduced ADSCs were aligned along DRGn axons for lengths that often exceeded 100 µm (Figure 9). Benztropine treatment increased both the number of O1^+^ cells and the width of their processes along axons. Confocal microscopy analysis showed O1 or MBP labelling surrounding βIII-tubulin- or neurofilament-labeled fibers, indicating that oligodendrocyte-like processes were ensheathing DRGn axons (Figures 9C,D).

**FIGURE 10:**
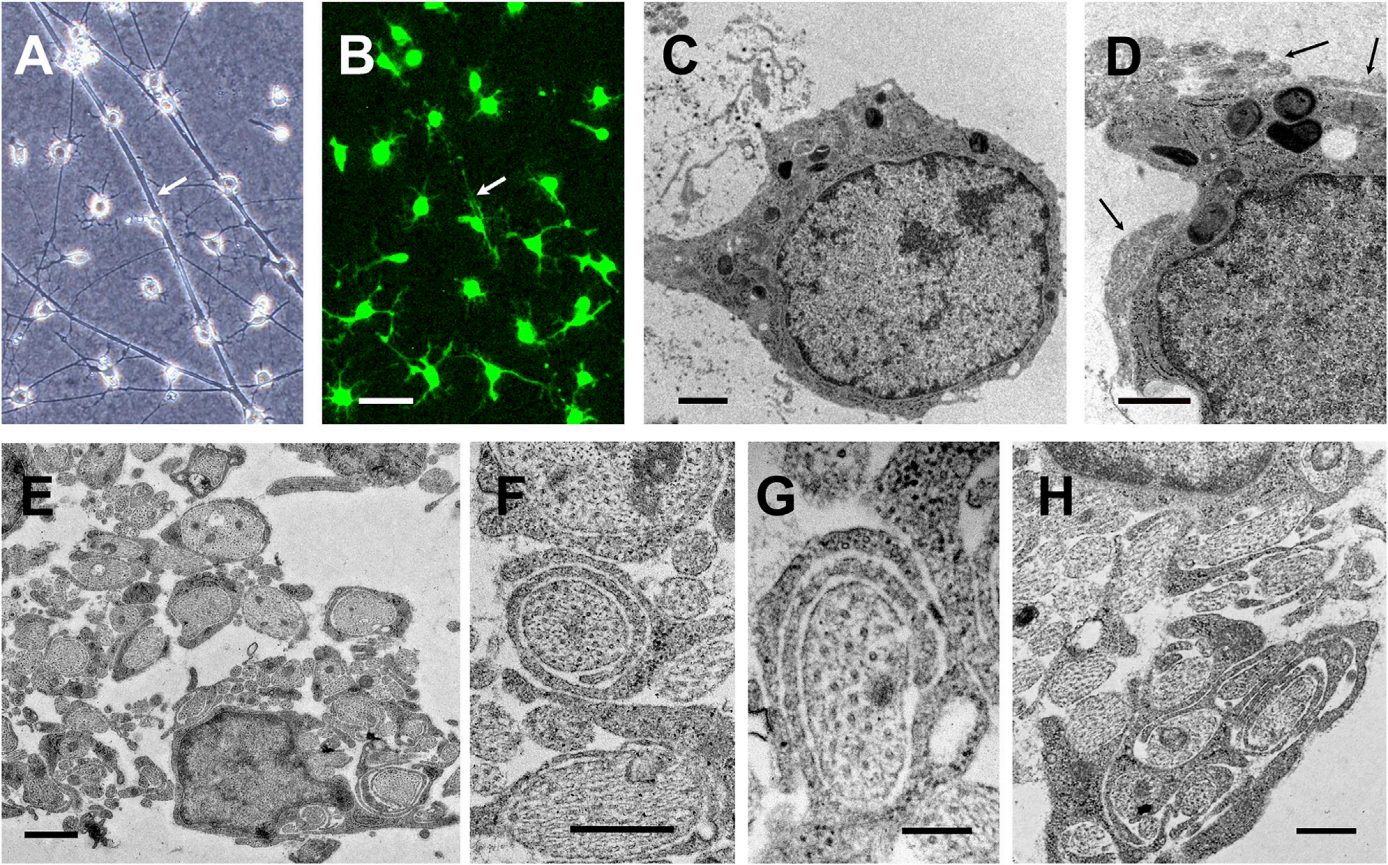
Axonal ensheathment by S + O + Z-transduced ADSCs in culture. Phase contrast **(A)** and fluorescence **(B)** images from the same field of DRG neurons co-cultured for 25 days with S + O + Z-transduced cells additionally transduced with green fluorescence protein; white arrows point to cytoplasmic extensions along axonal bundles (live cultures, scale bar: 50 µm). **(C–H)** Transmission electron micrographs of various co-cultures: **(C, D)** show converted cells with characteristic oligodendrocyte morphologies: dark nuclei occupying a large part of the cell body, with clumped chromatin beneath the nuclear membrane, and electron-dense cytoplasms that accumulate dark inclusions and extend several processes; these cells are usually found in close contact with axons (black arrows in **(D)**); no basal lamina was observed. Scale bar: 1 µm. **(E)** Multiple DRG neuronal axons are ensheathed by the dark processes of converted cells. Arrow points to an axon surrounded by several turns of converted oligodendroglial processes; scale bar: 1 µm. **(F, G)** Details of axons loosely ensheathed by several turns of converted oligodendroglial processes; scale bar in both images, 200 nm. **(H)** Axonal ensheathing in benztropine-treated co-culture. Scale bar: 500 nm.

TEM further demonstrated the generation of oligodendroglia-like cells among S + O + Z-transduced rat ADSCs. Converted cells showed dark nuclei occupying a large part of the cell body, with clumped chromatin beneath the nuclear membrane, and electron-dense cytoplasms that accumulate dark inclusions and extend several processes (Figures 10C,D). When seeded on areas of naked axons of DRGn cultures devoid of Schwann cells, converted cells extended processes that enveloped axon bundles and, in the case of larger axons (diameter > 0.5 µm), these processes enveloped them with one or more complete turns (Figures 10E–G). Additionally, benztropine-treated co-cultures showed enhanced ensheathment in some areas of the axonal network (Figure 10H). However, we were not able to consistently demonstrate the formation of compact myelin in these cultures.

Besides oligodendroglia-like cells, TEM indicated the presence of at least one more cell type in S + O + Z-transduced ADSC/DRGn co-cultures. These cells were larger than oligodendroglia, had a greater cytoplasm/nucleus ratio, and their nuclei were often highly indented, paler than those of oligodendroglia, and had a rim of heterochromatin near the nuclear membrane. Their cytoplasm was slightly dark (but paler than that of oligodendroglia) and contained an abundance of mitochondria, electron-lucent droplets, lysosomes, and ribosomes; bundles of filaments were also observed occasionally. Moreover, these cells contributed to the covering of axonal bundles with some cytoplasm extensions. Nevertheless, the ensheathment of individual axons with partial or complete turns was always carried out by electron-dense projections extending from oligodendroglia-like cells (see Supplementary Figures S16 and S17).

## 4 Discussion

In this study, we have demonstrated that adult rat ADSCs can be efficiently converted into oligodendrocyte precursor-like cells through the exogenous expression of genes encoding the transcription factors Sox10, Olig2, and Zfp536. These oligodendrocyte precursor-like cells were shown to be functional as they differentiated into mature (O1^+^) oligodendroglia, expressed glutamate and dopamine receptors, showed similar metabolic responses to OPCs, ensheathed axons, and showed similar responses to the same trophic factors and drugs as normal, nervous system-derived OPCs. Additionally, GFAP^+^ and p75NTR^+^ cells could be obtained from these S + O + Z-transduced ADSCs. Accordingly, the generated cells could more correctly be regarded as “induced macroglia.”

Adipose tissue appears to be a good source of cells for generating oligodendroglia by direct lineage conversion. It is accessible, can be easily and innocuously obtained from adults, and contains MSCs with neurotrophic (Joyce et al., 2010), neuroprotective, anti-inflammatory, and immunomodulatory properties (Yanez et al., 2006; De Miguel et al., 2012; Gebler et al., 2012), rendering them suitable candidates for use in cell transplantation.

In the growth conditions for converted cells used here, which included the supplementation of a defined medium with EGF, bFGF, and PDGF-AA, oligodendroglia-like cells were preferentially stimulated to proliferate and remained undifferentiated. This likely explains why these cells were progressively enriched in the cultures and outgrew non-converted cells. For the same reason, the proportion of O1^+^ cells (maturing oligodendrocytes) remained low despite the abundance of O4^+^ cells. Once the growth factors had been withdrawn, abundant O1^+^ cells were generated, and their development was enhanced with T_3_ supplementation.

### 4.1 Identification of S + O + Z-Transduced Cells

We used immunostaining with the O4 monoclonal antibody (Sommer and Schachner, 1981) as the initial marker for the generation of oligodendroglia from ADSCs. Many of the markers commonly used to identify oligodendroglia were already expressed by ADSCs growing in α20 medium, as evidenced by both immunofluorescence staining and RT-PCR (Vellosillo et al., 2017). MBP, MOG, DM20, CNPase, and GalC (as detected by O1 monoclonal antibody) (Sommer and Schachner, 1981), are spontaneously expressed in cultured ADSCs. However, these cells do not stain with anti-MAG or with the O4 antibody, which recognizes sulfatides in the membranes of OPCs (Bansal et al., 1989). Consequently, we considered these two labels to be specific markers for the conversion of ADSCs into oligodendroglia-like cells.

Chondroitin sulfate proteoglycan 4, detected by the NG2 antibody, is often used to identify OPCs in the CNS. Although different terms have been used to describe these precursor cells, they could simply be called “NG2-glia” because they differentiate into oligodendrocytes or astrocytes, depending on their location in the brain (Dimou and Gallo, 2015). In our study, we could not use NG2 as a marker of oligodendroglial generation, even though it was expressed in S + O + Z-transduced ADSCs, as more than 40% of rat ADSCs were already NG2^+^ when cultured under basal conditions (Vellosillo et al., 2017). This was also true for another OPC marker, PDGFRα, which has been shown to be expressed by at least a subset of MSCs (Mohsen-Kanson et al., 2013).

The A2B5 antibody could potentially also serve as a marker for identifying glial progenitor cells. This monoclonal antibody defines bipotential oligodendrocyte-type 2 astrocyte progenitor cells in CNS cultures and is regularly used to identify glial precursors that differentiate into oligodendrocytes under the appropriate culture conditions. However, this antibody will also bind to unidentified glycoproteins in the cytoskeleton and mitochondria when cells are permeabilized during the immunostaining procedure (Gillard et al., 1993), potentially resulting in intracellular staining and false positives. Irrespective of this possibility, no membrane labelling with A2B5 was detected in S + O + Z-transduced cultures. This result suggests that the conversion process induced by exogenous *Sox10*, *Olig2*, and *Zfp536* expression in ADSCs does not require such developmental stage.

Interestingly, GFAP^+^ cells were generated during the S + O + Z-induced conversion of rat ADSCs, an effect that was greatly enhanced when the culture was supplemented with 10% FBS. GFAP is a marker for several glial cell types, such as astroglia, subventricular zone neural stem cells (Doetsch et al., 1999), olfactory ensheathing cells (Ramon-Cueto and Nieto-Sampedro, 1992), non-myelinating Schwann cells (Mokuno et al., 1989; Feinstein et al., 1992), and enteric glia (Jessen and Mirsky, 1980), in addition to several non-neural cell types. The generation of GFAP^+^ cells resulting from the expression of exogenous *Sox10*, *Olig2*, and *Zfp536* has also been reported in embryonic fibroblasts (Yang et al., 2013); however, studies employing different induction strategies have not obtained similar results (Najm et al., 2011; Garcia-Leon et al., 2018).

The identity of the converted cells that expressed p75NTR, a low-affinity NGF receptor, requires further investigation. p75NTR is expressed by different cell types in the peripheral nervous system (PNS) and CNS. It is typically expressed in neural crest stem cells (Stemple and Anderson, 1992), non-myelinating Schwann cells (DiStefano and Johnson, 1988), and olfactory ensheathing glia (Ramon-Cueto and Nieto-Sampedro, 1992); accordingly, this receptor has been used for the immunoselection of these cell types in culture. Additionally, several studies have reported the existence of a population of p75NTR^+^ cells, termed “CNS Schwann cells” by the authors, that originate from a common CNS precursor cell population with OPCs and remyelinate axons after a demyelinating insult (Keirstead et al., 1999; Zawadzka et al., 2010). These “CNS Schwann cells” share characteristics with OPCs that distinguish them from PNS Schwann cells (Kegler et al., 2014). In our experiments, the p75NTR^+^ cells detected in converted cultures had an elongated but branched morphology that differed from the morphology of typical PNS Schwann cells in culture*.* Notably, however, S + O + Z-transduced ADSCs also exhibited *de novo* mRNA expression of at least two Schwann cell-specific proteins, periaxin and myelin protein zero (MPZ), primarily when cultures were maintained in FBS-supplemented medium (D10; see Supplementary Figure S12), which supports their identity as Schwann cells.

This notwithstanding, some differentiated converted cells co-expressed markers such as O4, GFAP, and p75NTR (Figure 4), and we identified a cell population that shows ultrastructural characteristics (a less electron-dense cytoplasm; abundant mitochondria, ribosomes, and lysosomes; and a nucleus with a peripheral rim of condensed chromatin; Supplementary Figures S16B and S17) similar to those seen in olfactory ensheathing glia (Ramon-Cueto and Avila, 1998; Gomez et al., 2016). Further studies are required to determine whether S + O + Z-transduced ADSCs can generate olfactory ensheathing glia-like cells, and whether such cells exhibit similar neurotrophic properties.

In this study, S + O + Z-transduced cells expressed mRNAs of myelin-related molecules such as MBP, PLP1, MOG, and MAG. The expression of these proteins was greatly stimulated after growth factor withdrawal and medium supplementation with T_3_ (Figure 3, see also Supplementary Figures S8 and S10)*.* In neural tissues, MBP is expressed as different isoforms with roles in both oligodendrocyte development and myelin compaction (Harauz and Boggs, 2013), while the expression of the PLP1 isoform and MOG has been associated with oligodendrocyte maturity. MAG, however, was one of the earliest molecules expressed in S + O + Z-transduced cells. This is particularly surprising given that, in both the PNS and CNS, MAG begins to be expressed at the initial steps of myelination, simultaneously with MBP (Dubois-Dalcq et al., 1986). Indeed, in large oligospheres, which showed some degree of glial differentiation and high GFAP expression, both MAG and MBP were weakly expressed, as shown in Figure 5. In contrast, both L- and S-isoforms of MAG, which are expressed early and late in CNS myelination, respectively (Ishiguro et al., 1991; Pedraza et al., 1991), were found to be strongly expressed already by day 24 following the induction of *Sox10*, *Olig2*, and *Zfp536* transgene expression (Figure 5C).

QKI-7, the marker detected by the CC1 monoclonal antibody (Bin et al., 2016), is widely used for identifying myelinating oligodendrocytes. S + O + Z-transduced ADSCs expressed high levels of *Qki7* mRNA under conditions that support expansion (Figure 2J). Quaking proteins are key regulators of the metabolism of transcription factor RNA and the expression of myelin-related genes involved in oligodendrocyte differentiation (Darbelli et al., 2017). The expression of *Qki7* in converted cells (but not in untransduced ADSCs) is strong evidence supporting that the *Sox10*, *Olig2*, and *Zfp536* transgene combination induces the generation of oligodendroglial cells.

Similarly, both Cx29 and Cx47 were expressed *de novo* in converted cells. That these two connexins are specific to oligodendroglia provides a clear demonstration of the acquisition of an oligodendroglial phenotype and oligodendroglial functionality in S + O + Z-transduced ADSCs. The expression of oligodendroglial-specific connexins has not been previously reported in studies relating to the generation of oligodendrocytes through reprogramming or direct conversion despite their functional relevance. Oligodendrocyte connexins are known to play an important role in axonal ionic and metabolic homeostasis (Rash et al., 2016; Fasciani et al., 2018) and are also required for the maintenance of myelination (Uhlenberg et al., 2004; Orthmann-Murphy et al., 2007).

### 4.2 Minimal Requirements for Oligodendroglia-Like Cell Generation From Adult ADSCs

The expression of the *Sox10* transgene alone stimulated endogenous *Olig2* and *Zfp536* expression (Figures 6A,B), but not at sufficient levels to support oligodendroglia-like cell generation from adult rat ADSCs. This was despite the fact that the O4 labeling and the mitogenic response to EGF, bFGF, and/or PDGF-AA were induced under the same conditions. Furthermore, as the *Olig2* and *Zfp536* transgenes did not stimulate endogenous *Sox10* expression, the inclusion of the *Sox10* transgene appears to be required for generating oligodendrocyte precursor-like cells from adult rat ADSCs. The *Sox10* + *Olig2* combination did not appear to be sufficient for generating oligodendroglia-like cells from adult rat ADSCs given that it did not stimulate a complete morphological conversion or the appearance of MAG^+^ cells. Additionally, this combination did not induce the generation of GFAP^+^ cells, which might be required for OPC maintenance and differentiation.

### 4.3 Transcription Factor Combinations for Generating Oligodendroglia May Differ Among Species

Exogenously expressing *Sox10*, *Olig2*, and *Zfp536* to generate oligodendroglia-like cells from adult rat ADSCs may not be directly extrapolated to other species. We have found that efficiently converting human ADSCs into oligodendroglia-like cells using the methodology reported here may require different transgene combinations and/or additional molecules (see, for instance, Supplementary Figure S18). It has also been reported that the direct conversion of embryonic fibroblasts from mice and rats into oligodendroglia might require different sets of transcription factors (Lee and Park, 2017).

In addition to species, other variables might influence which transcription factor combinations can be used for converting somatic cells into oligodendroglia. The cell type and/or developmental stage of the donor appear to be particularly relevant (Najm et al., 2013) reported that a combination of *Sox10* + *Olig2* + *Nkx6.2* was sufficient to generate functional oligodendroglia from E13.5 embryonic mouse fibroblasts. In the present study, we were unable to generate oligodendrocyte precursor-like cells from adult rat ADSCs using a similar combination (*Sox10* + *Olig2* + *Nkx6.1*). Furthermore, when *Nkx6.1* was added to the *Sox10* + *Olig2* + *Zfp536* combination, few oligodendrocyte precursor-like cells were obtained unless the cultures were pre-treated with RA, which altered cell morphology and composition. Interestingly, when the *Sox10* + *Olig2* + *Nkx6.2* combination was used for the conversion of embryonic mouse fibroblasts, no GFAP^+^ cells were generated, in contrast to that seen with the *Sox10* + *Olig2* + *Zfp536* combination, as shown in the present study and elsewhere (Yang et al., 2013). Sox10 appears to be the key factor for oligodendroglial commitment (Yang et al., 2013) while Olig2 is a common element in all lineage conversions into oligodendroglia, including that of human iPSCs (Pawlowski et al., 2017). Of note, the time required for OPC generation when embryonic cells (or iPSCs) are used is considerably shorter compared with that when adult ADSCs are converted.

### 4.4 The *Sox10*, *Olig2*, and *Zfp536* Transgenes Must Be Continuously Expressed for the Maintenance of the Oligodendroglial-Like Phenotype

The converted cells required the continuous expression of the *Sox10*, *Olig2*, and *Zfp536* transgenes to maintain the oligodendroglial-like cell phenotype as well as the ability to ensheathe axons. The withdrawal of doxycycline for 14 days resulted in phenotype reversion, including the lack of mitogenic response to the growth factors EGF, bFGF, and PDGF-AA, as well as decreased expression of endogenous *Olig2* and *Zfp536* and the loss of myelin-related proteins (Figures 6C,D). Even after 6 months of continuous *Sox10* + *Olig2* + *Zfp536* transgene expression, with O4^+^ cells comprising >95% of the cells in culture, doxycycline withdrawal resulted in the loss of the oligodendroglial phenotype.

Accordingly, the direct conversion of ADSCs into glial cells through exogenous *Sox10* + *Olig2* + *Zfp536* expression cannot be considered “lineage commitment” or “reprogramming” as the process is reversible. This further suggests that, unlike that observed when cells are reprogrammed to pluripotency, the epigenetic traits that maintain the phenotype of the original cells are not completely replaced when the new transcription factors drive the emergence of oligodendroglial characteristics. Nevertheless, cells cultured in a dish lack the necessary environmental cues that normally shape their identity, and it remains to be investigated whether maintaining these oligodendrocyte precursor-like cells in a complex neural environment, where they can receive three-dimensional functional signals (axons, morphogens, trophic factors, etc.), would result in complete and irreversible phenotypic conversion. The continuous *Sox10* + *Olig2* + *Zfp536* transgene expression, however, did not prevent the development of a mature oligodendroglial phenotype.

### 4.5 The Functionality of the Generated Oligodendrocytes

We have provided several evidences to support that S + O + Z-transduced ADSCs function as oligodendroglia. We showed that, when co-cultured with DRGns, the oligodendroglia-like cells produced axon-ensheathing processes. We further demonstrated that a given converted cell could ensheathe several axons from different neurons in the absence of basal lamina (Figure 9), as occurs with regular CNS oligodendrocytes.

We also detected the mRNA expression of AMPA, NMDA, and kainate subunits of glutamate receptors in these oligodendroglia-like cells. Even when their expression levels were low, it was likely to have been *de novo* expression, as unconverted ADSCs maintained under basal conditions did not express these receptors (Figure 7; Supplementary Figure S13). Whether receptor agonists or other treatments can stimulate the expression and activity of glutamate receptors in converted cells merits further investigation. Dopamine receptors have also been reported to be present in OPCs, although relevant studies are scarce (Bongarzone et al., 1998). Our results corroborate previous, unpublished observations that NSC-derived oligodendroglial cultures express D1-type receptors (DR1 and DR5) and the DR2 subtype of D2-type receptors. Importantly, S + O + Z-transduced ADSCs also expressed DR1, DR2 and DR5 mRNA. Expression of dopamine receptors by S + O + Z-transduced ADSCs further supports their functional capacities as oligodendroglia.

Our study further demonstrated that adult rat ADSC-derived oligodendroglia-like cells are neurochemically functional as they respond to pharmacological stimuli that drive the maturation and myelinating capacity of CNS-derived oligodendrocytes (Deshmukh et al., 2013). In co-cultures of DRGns with converted cells, the addition of benztropine, a muscarinic antagonist that has been repurposed as a stimulator of OPC maturation and inducer of myelination, led to the generation of larger numbers of O1^+^ cells as well as more profuse axonal ensheathment (Figure 9).

We also investigated the mitochondrial function profile in converted cells and found that it was similar to that of NSC-OPCs. The results of several studies have suggested that OPC differentiation is accompanied by an increase in mitochondrial function and that the myelination process induces metabolic changes that favor the synthesis of myelin lipids (Rone et al., 2016; Tepavcevic, 2021). Myelination is an energy-demanding process. Several metabolites and drugs (benztropine, metformin, taurine) promote OPC differentiation by enhancing the mitochondrial respiratory capacity. Our data showed that benztropine treatment led to a substantial increase in the basal and maximal respiratory index in both NSC-OPCs and S + O + Z-transduced ADSCs, but particularly in the latter (Figure 8).

TEM analysis (Figure 10) showed large axons surrounded by one or several turns of processes emanating from converted oligodendroglia-like cells, which might provide metabolic support to those axons. The expression of the connexins Cx47 and Cx29 in S + O + Z-transduced cells, which would enable the intercellular flow of ions and small metabolites such as lactate or pyruvate (Philips and Rothstein, 2017), supports this hypothesis.

However, we failed to consistently demonstrate the presence of compact myelin around axons in our *in vitro* model, which may be related to technical or artefactual complications involving the culture conditions used. Consequently, it remains unknown whether these converted ADSCs have the capacity to myelinate *in vivo* when transplanted into animals. In embryonic fibroblasts, the expression of the *Sox10* + *Olig2* + *Zfp536* transgene combination generates cells capable of forming compact myelin when transplanted into the brain or spinal cord (Yang et al., 2013), suggesting that our converted adult rat ADSCs might also have this capacity. However, the balance between MBP and CNPase reportedly determines which membrane regions are compacted and which retain cytoplasm and serve as channels for communication between peripheral and somatic zones of myelinating oligodendrocytes (Snaidero et al., 2017). Because ADSCs express CNPase under basal conditions, as shown in Figure 5 and in a previous study (Vellosillo et al., 2017), which does not appear to be the case for embryonic fibroblasts (Yang et al., 2013), a high CNPase-to-MBP ratio in ADSC-derived oligodendroglia might explain the lack of myelin compaction in our experiments.

### 4.6 Similarities Between Oligodendroglia Converted From ADSCs and Those Derived From Neural Stem Cells

Besides morphological similarities, cells converted from adult rat ADSCs through *Sox10* + *Olig2* + *Zfp536* transgene expression showed the same molecular signature and responded to the same trophic factors (EGF, bFGF, and PDGF-AA) as neural-derived oligodendroglia. They were also similarly stimulated to differentiate by T_3_ or benztropine supplementation, and also displayed a high affinity for attaching to and ensheathing axons. Regarding differences, compared with neural OPCs, S + O + Z-transduced ADSCs display very low endogenous *Sox10* expression, lower levels of several oligodendroglial genes, immunostaining for MAG before differentiation, and growth in culture as plastic-adherent cells instead of as floating aggregates (oligospheres).

Additionally, as mentioned above, S + O + Z-transduced ADSCs are not fully reprogrammed and may retain characteristics and properties of the original ADSCs. *In vivo* experiments using animal models of demyelination should be undertaken to determine if these converted cells can indeed efficiently remyelinate nude axons, as occurs with neural-derived OPCs (Groves et al., 1993; Archer et al., 1997; Learish et al., 1999), and also if their vestigial mesenchymal traits represent advantages or disadvantages regarding their potential use for cellular therapy in demyelinating diseases.

Given these observations, although we initially considered S + O + Z-transduced ADSCs to be “induced OPCs,” the differences between them and neural-derived OPCs suggest otherwise. Nevertheless, many of the characteristics displayed by these converted cells support that they behave like oligodendroglial cells. Moreover, when these cultures were maintained in 10% FBS-supplemented medium (D10 medium, for instance), the generated cells were comparable to astroglia and, putatively, also to Schwann cells/olfactory ensheathing glia-like cells. Thus, although our original goal was to generate oligodendroglia from peripheral tissues, the present study showed that S + O + Z-transduced ADSC can also be converted to other macroglial types.

### 4.7 Limitations of the Method

As discussed above, the direct conversion of adult rat ADSCs into oligodendroglia has some limitations. Cells generated through *Sox10* + *Olig2* + *Zfp536* transgene expression displayed most of the phenotypic, molecular, pharmacological, differentiating, and functional characteristics of brain oligodendroglia, but are not exactly the same cell type. Although the converted cells acquired a new phenotype, they retained at least some of their former ADSC identity, and continuous transgene expression was required to maintain the oligodendroglial phenotype.

Another limitation of this study was that, at present, the procedure cannot be directly extrapolated to human cells although, as mentioned above, we also hypothesize that generating oligodendroglia by direct conversion from adult human ADSCs is possible once the appropriate transgene combination and culture conditions have been identified. On the other hand, the direct conversion of adult somatic cells into oligodendroglia takes longer than conversion of embryonic cells, even when using MSCs. In rats, the procedure becomes efficient and leads to the production of large numbers of oligodendroglia-like cells after 2–3 months; however, this will likely take longer in adult humans.

Regarding methodological limitations, the RT-qPCR results showed high variability, which affected the statistical significance of differences between source ADSCs and S + O + Z-transduced cells. Accordingly, non-parametric tests and data pairing for treatment conditions of sibling cultures were used when comparing groups whenever possible. Data variability did not appear to originate from technical issues associated with the use of a single housekeeping reference gene (*Gapdh*) since a triple housekeeping reference that included *Rpl13a* and *Ppia* did not improve variability compared with the use of *Gapdh* alone (data not shown). Instead, mRNA expression patterns depended on when S + O + Z-transduced cell samples were processed. We regularly used cultures at 2–4 months of transgene expression, when between 30% and 70% of O4^+^ cells had been generated (Figure 2C). Despite the consistency in ADSC conversion through *Sox10* + *Olig2* + *Zfp536* transgene expression, the values of mRNA expression were variable within a wide range. Analysis of the effects of different treatments on these converted cells might benefit from O4^+^ cell selection as starting material. Meanwhile, NSC-derived oligodendroglia were used as a positive control to determine the similarities between S + O + Z-transduced ADSCs and neural oligodendroglia. This was not meant to provide quantitative comparison of gene expression between the 2 cell types as NSC-derived oligodendroglia culture consists of 75%–95% oligodendroglial-committed cells, which might not be comparable to other oligodendroglial cell preparations. Indeed, there is no prototypical oligodendroglial cell culture. Accordingly, we chose to use NSC-derived cultures as positive controls mainly due to their consistency in generating oligodendroglial-committed cells.

### 4.8 Alternatives for Generating Oligodendroglia From Adult Somatic Cells

Oligodendroglia can be generated by reprogramming adult somatic cells to pluripotency and then committing these iPSCs to a neural precursor cell type. OPCs are subsequently generated from the latter by complex cell culture procedures and the use of media that include specific morphogens, growth factors, and small molecules that either inhibit or activate intracellular mechanisms (Wang et al., 2013; Douvaras and Fossati, 2015; Espinosa-Jeffrey et al., 2016; James et al., 2021). The advantages of these procedures are that they are suited to human cells and generate oligodendroglia in which epigenetic traits of the source cells have been erased. The disadvantages are the protracted time required to obtain the first oligodendrocytes (>3 months from the start of somatic cell culture) and that the complexity of the method may impair its consistency. Besides oligodendroglia, these cultures also generate astroglia and neurons. The complexity and prolonged duration associated with this strategy can be slightly improved by transducing iPSCs-derived NSC with a combination of *SOX10*, *OLIG2*, and *NKX6.2* (Ehrlich et al., 2017), *SOX10* and *OLIG2* (Pawlowski et al., 2017), or only *SOX10* (Garcia-Leon et al., 2018) transgenes.

The generation of oligodendrocyte progenitor cells from adult mouse skin fibroblasts (Kim et al., 2015), human fibroblasts (Mitchell et al., 2014), or human blood cells (Lee et al., 2015) *via Oct4* transgene expression has been reported. Oct4, a transcription factor, is a key regulator of embryogenesis and has been found to be sufficient for inducing pluripotency (Kim et al., 2009). The direct conversion of adult somatic cells to oligodendroglia through Oct4 expression relies on the composition of the culture medium and, in particular, on the spontaneous silencing of the *Oct4* transgene after a few weeks; otherwise, as Oct4 can promote the generation of neural stem cells from human fibroblasts (Mitchell et al., 2014) and iPSCs from neural stem cells (Kim et al., 2009), the final product would be iPSCs, which could lead to the generation of teratomas when these cells are transplanted. Thus, because of the requirement for the precise control of Oct4 dosage (Niwa et al., 2000), inducing oligodendroglia from adult somatic cells by *Oct4* transfection may result in inconsistent results between research laboratories.

Pharmacological reprogramming of mouse embryonic and postnatal fibroblasts using chemically defined conditions, without genetic manipulation, has also been reported (Zhang et al., 2016). The authors used a combination of nine small molecules to induce fibroblasts to a neural program, and then used another combination of chemicals in selected cells to obtain oligodendrocyte- and astrocyte-like cells (Liu et al., 2019). Whether these chemically-induced oligodendrocytes are stable and have the capability of forming compact myelin remains to be determined.

### 4.9 Adult ADSCs as a Source of Oligodendroglia

ADSCs have been shown to display trophism, and can protect and repair CNS myelin even when intravenously administered (Otero-Ortega et al., 2015). Such regenerative capacity appears to be due to the activity of released molecules or extracellular vesicles (Otero-Ortega et al., 2017). Additionally, ADSCs have been shown to enhance remyelination in animal models of neuroinflammation through multiple mechanisms (Constantin et al., 2009) and, like MSCs, show a preference for homing to sites of injury/inflammation (Ullah et al., 2019). Consequently, ADSCs are considered to be potential candidates for use in cellular therapy in demyelinating diseases, particularly multiple sclerosis.

Here, we showed that, in addition to oligodendroglial phenotypes, S + O + Z-transduced adult rat ADSCs maintained some characteristics of their parent cells, suggesting that they may also retain some of the trophic, immunomodulatory, anti-inflammatory, and homing properties of ADSCs. Although additional studies are needed to confirm this possibility, it can be hypothesized that both locally and systemically delivered S + O + Z-transduced ADSCs may provide remyelination in experimental allergic encephalomyelitis models of demyelination, overcoming the autoimmune/inflammatory environment.

In summary, we have shown that oligodendroglia-like cells can be generated by direct conversion from adult rat ADSCs. The expression of transgenes encoding the Sox10 + Olig2 + Zfp536 transcription factors was found to be an effective procedure for generating induced macroglial cells. Oligodendroglia-like or astroglia/Schwann-like cells were preferentially generated, depending on the culture conditions. Additionally, S + O + Z-transduced ADSCs in co-culture with neurons were able to ensheathe axons.

In the present study, we demonstrated that oligodendroglia-like cells can be generated from adult rat peripheral tissues. ADSC-derived oligodendroglia might be a safe and convenient alternative for cell therapy strategies in demyelinating diseases. An efficient combination of transcription factors for generating oligodendroglia from adult human peripheral tissues without going through an iPSC stage has yet to be identified; nevertheless, our study supports the feasibility of such an approach.

## Data Availability

The original contributions presented in the study are included in the article/Supplementary Material, further inquiries can be directed to the corresponding author.
