# Supplementary material for: Oligodendroglia Generated From Adult Rat Adipose Tissue by Direct Cell Conversion
Source: Front Cell Dev Biol. 2022 Feb 11;10:741499. doi: 10.3389/fcell.2022.741499 (PMC8873586; doi:10.3389/fcell.2022.741499)
Supplement: Supplementary file 1 [file DataSheet1.PDF]

Supplementary material

1 Supplementary Figures and Tables

1.1 Supplementary Figures

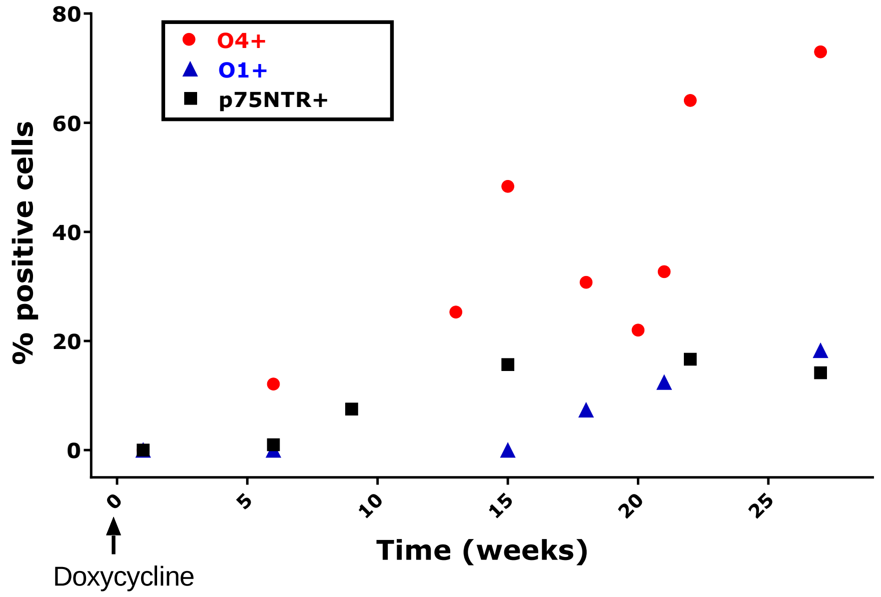

Supplementary Figure 1 **Time course of the immunophenotype in one experiment of Sox10 + Olig2 + Zfp536-transduced cells followed for 28 wk after transgene induction by doxycycline**. At the end of this experiment, more than 70% of cells were $O4^+$, 18 % were $O1^+$ and 14 % were $p75NTR^+$ (low-affinity NGF receptor). Double labelling studies for these immunomarkers were not done because these three monoclonal antibodies were of mouse origin, but it can be expected that at least some cells co-express these markers. The cells were continuously proliferating along the tested period and were passaged when showing signs of cell aggregation, usually every 1-2 wk, at which moment some coverslips were seeded for immunocytochemical studies or experiments. In the present case, after 7 months the cells suddenly underwent apoptosis. In some experiments, $O4^+$ cells constituted more than 90% of the cells in the culture.

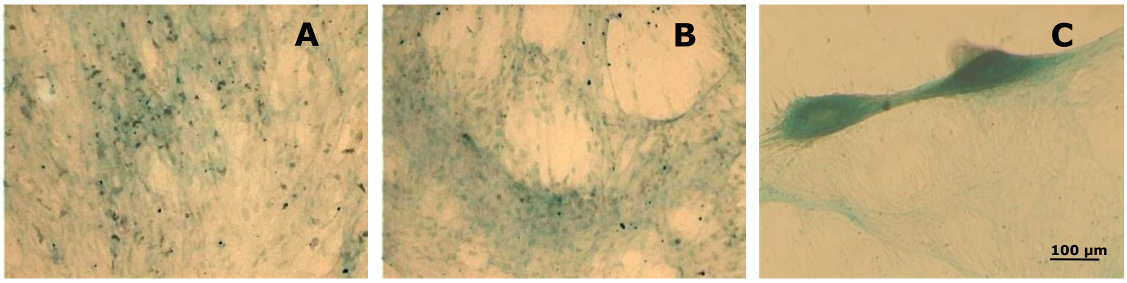

Supplementary Figure 2 **Alcian blue staining of glycosaminoglycans indicating chondrogenic differentiation of adult rat ADSCs after 3 weeks** in the following conditions: **A**: DMEM + 10 % FBS. **B**: Chondrogenic cocktail (DMEM + 10 % FBS + 10 ng/ml TGF-β1 + 150 nM phospho-L-ascorbic + 10 ng/ml insulin + 100 nM dexamethasone) **C**: Sox10 + Olig2 + Zfp536-transduced ADSCs in chondrogenic cocktail. The chondrogenic cocktail stimulated cell aggregation and glycosaminoglycan expression (stained in blue). When cells overexpressed the transcription factors, chondrogenic differentiation was enhanced. Since this tendency for chondrogenesis in Sox10 + Olig2 + Zfp536-transduced cells could be competing or delaying the lineage conversion of ADSCs into oligodendroglial-like cells, diverse pharmacological pre-treatments that would inhibit ADSC chondrocytic differentiation were tested

---

(next page)

Supplementary Figure 3. **Morphological and phenotypical differences of the transgene expression of Sox10 (S), Sox10 + Olig2 (S + O) or Sox10 + Olig2 + Zfp 536 (S + O + Z) in rat ADSC after 10 weeks**. Phase contrast images show that S transgene expression (A_1) is not accompanied by a refringent and branching morphology of cells. S + O induce some branching (B_1) but cells are still far from the typical oligodendroglial morphology attained by S + O + Z overexpression (C_1). Sox10 alone induces the expression of the O4 antigen (A_2) but the cells are not branched and MAG^+ cells are rarely found (not shown). $O4^+$ cells in S + O-transduced cells display more branching (B_2) and the sporadic MAG^+ cells show oligodendrocyte-like morphology. Expression of the full set of transgenes, (S + O + Z) produces typical $O4^+$ and show numerous MAG^+ cells of oligodendroglial morphology. Scale bar: 100 µm for phase contrast images; 50 µm for immunofluorescent images.

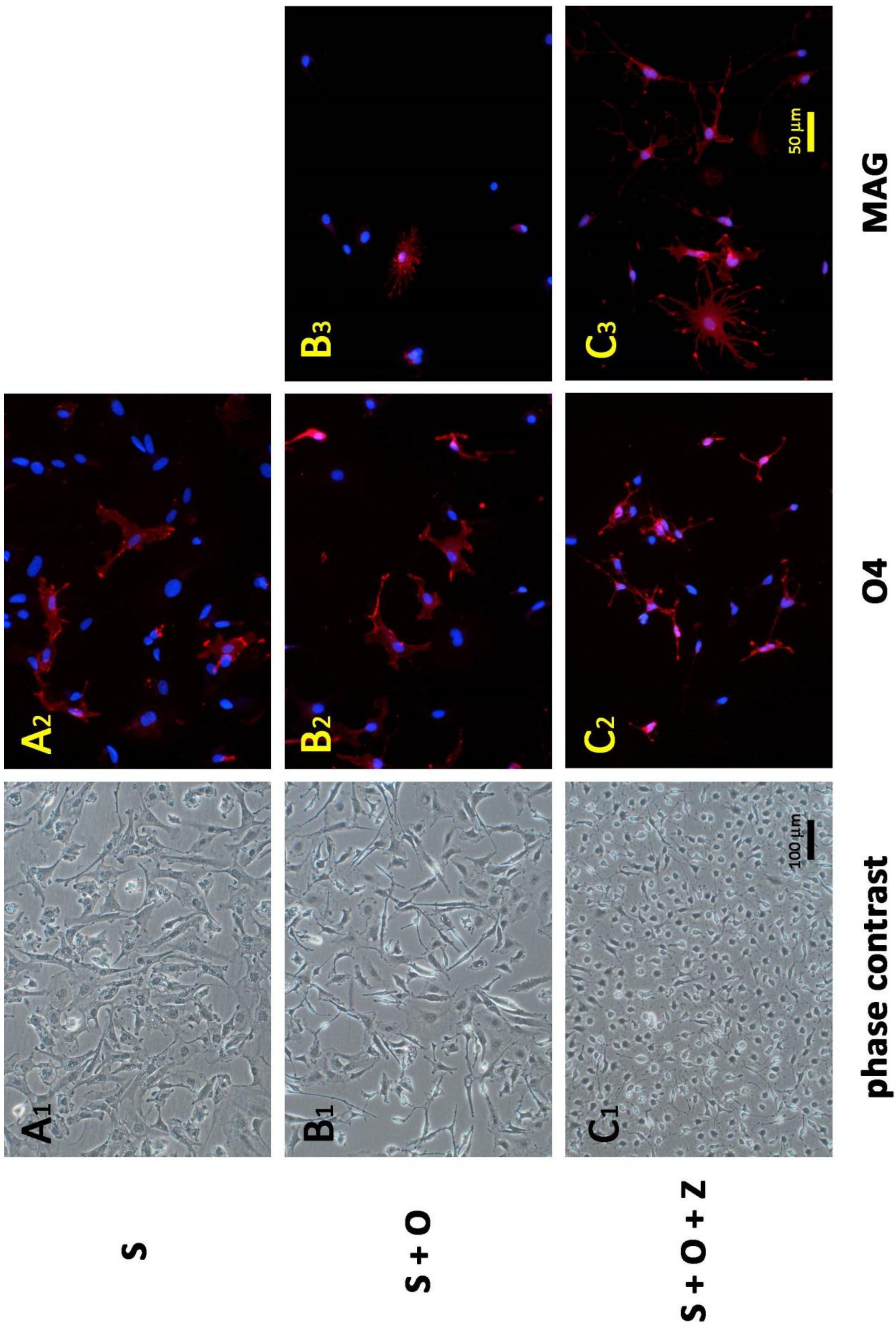

Supplementary Figure 3

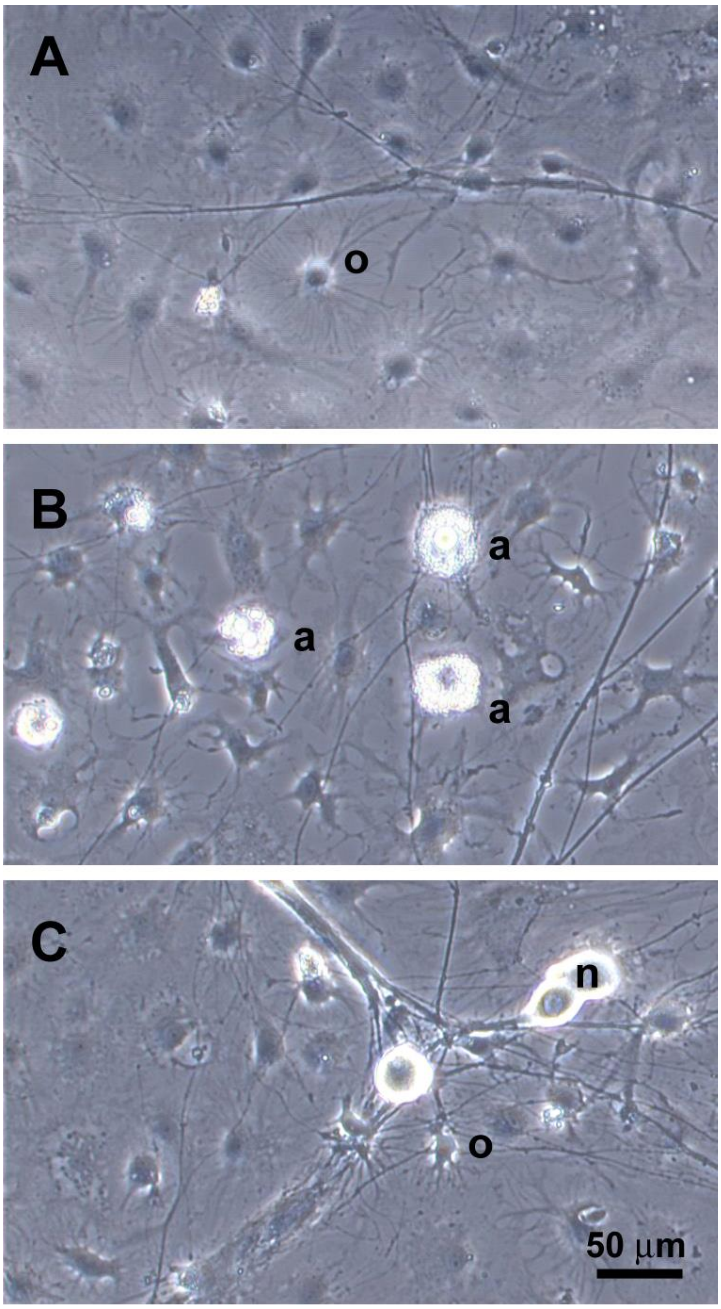

<u>Supplementary Figure 4</u> **Phase contrast image of co-cultures of dorsal root ganglion neurons with adult rat ADSCs expressing S + O + Z transgenes for 10 weeks** under the control of a Tet-ON conditional promoter. In **A**, when the cultures are maintained in doxycycline-containing medium, some cells show oligodendrocyte (O) morphology and relate to axons. In **B**, when doxycycline is withdrawn from the medium, most cells change their morphology and some of them become lipid droplet-containing adipocytes (a). In **C**, if doxycycline is returned to the feeding medium, adipocytes disappear and small branched, axonal-related oligodendrocyte-like cells show up again in 1 week. (n): DRG neuronal bodies.

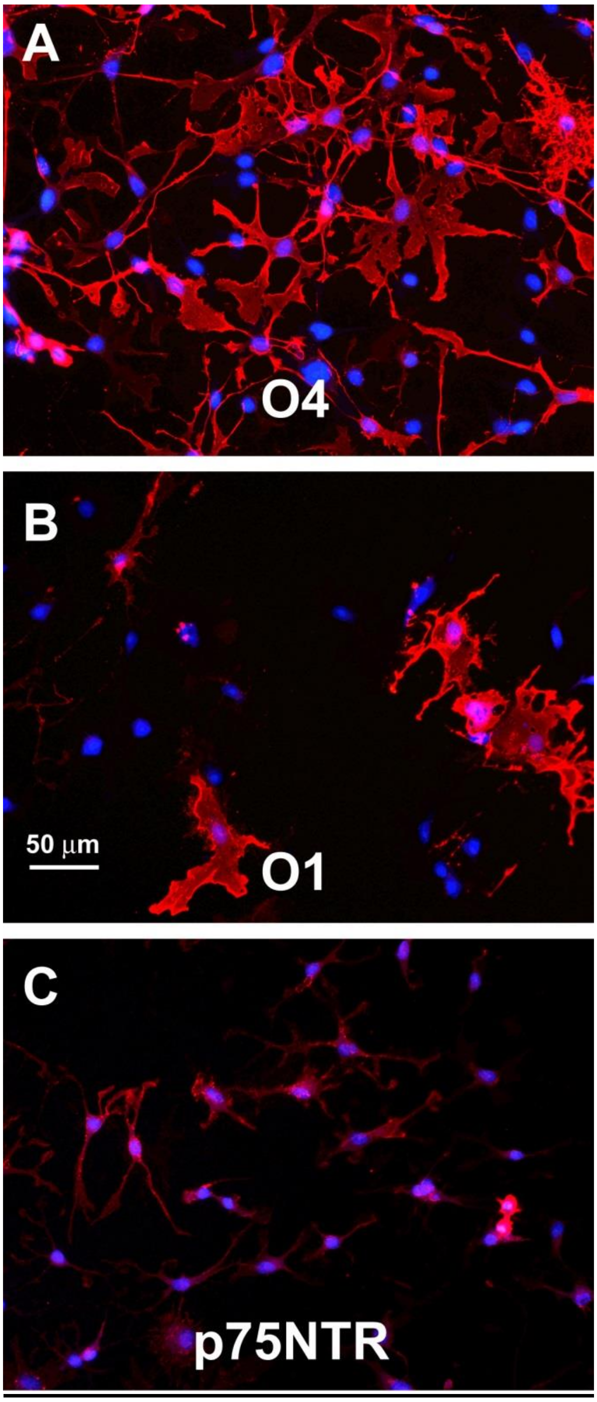

Supplementary Figure 5. **Glial markers of S + O + Z + N-transduced cells that had been pre-treated with all-trans retinoic acid 1 µM for 5 days before transgene activation with doxycycline**. After 3 months, cultures showed $O4^+$ cells, but their morphology was not typical of OPCs (A). Fewer cells were positive for O1 and also displayed aberrant morphologies (B). Numerous cells showed expression of p75NTR (C). Scale bar for all pictures: 50 µm

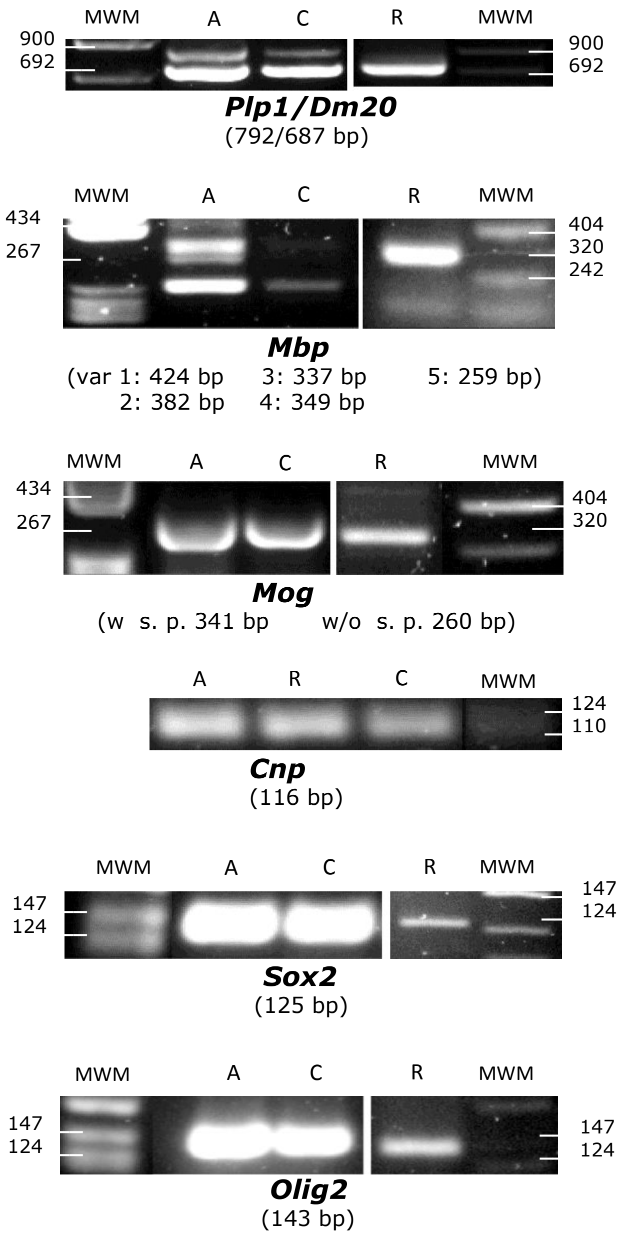

<u>Supplementary Figure 6.</u> **Effect of pre-treatments on the expression of oligodendroglial-related molecules in S + O + Z-transduced adult rat ADSCs**. End-point RT-PCR showing mRNA expression of various myelin-related molecules (*Plp1/Dm20*, *Mbp*, *Mog*, *Cnp*), as well as the transcription factors *Sox2* and *Olig2* (endogenous) in cells in which the transgenes had been induced without any pre-treatment (C), or after treatment with adipogenic cocktail (A) or all-trans retinoic acid (R). Amplicon sizes obtained with our primers are specified between parentheses. Using these primers, more than one isoform can be distinguished for *Plp1/Dm20* (*Plp1* and *Dm20* isoforms), *Mbp* (non-Golli variants 1 to 5) and *Mog* (with or without signal peptide). MWM: molecular weight marker. Notice that adipose pre-treatment for 5 days prior to transgene induction may change the mRNA expression of the different isoforms of myelinating proteins *Plp*1 and *Mbp*. Retinoic acid pre-treatment produced less mature isoforms

Note: The RT-PCRs here shown were performed in different days (except for Cnp) and, therefore, labelling intensity is not comparable in all cases.

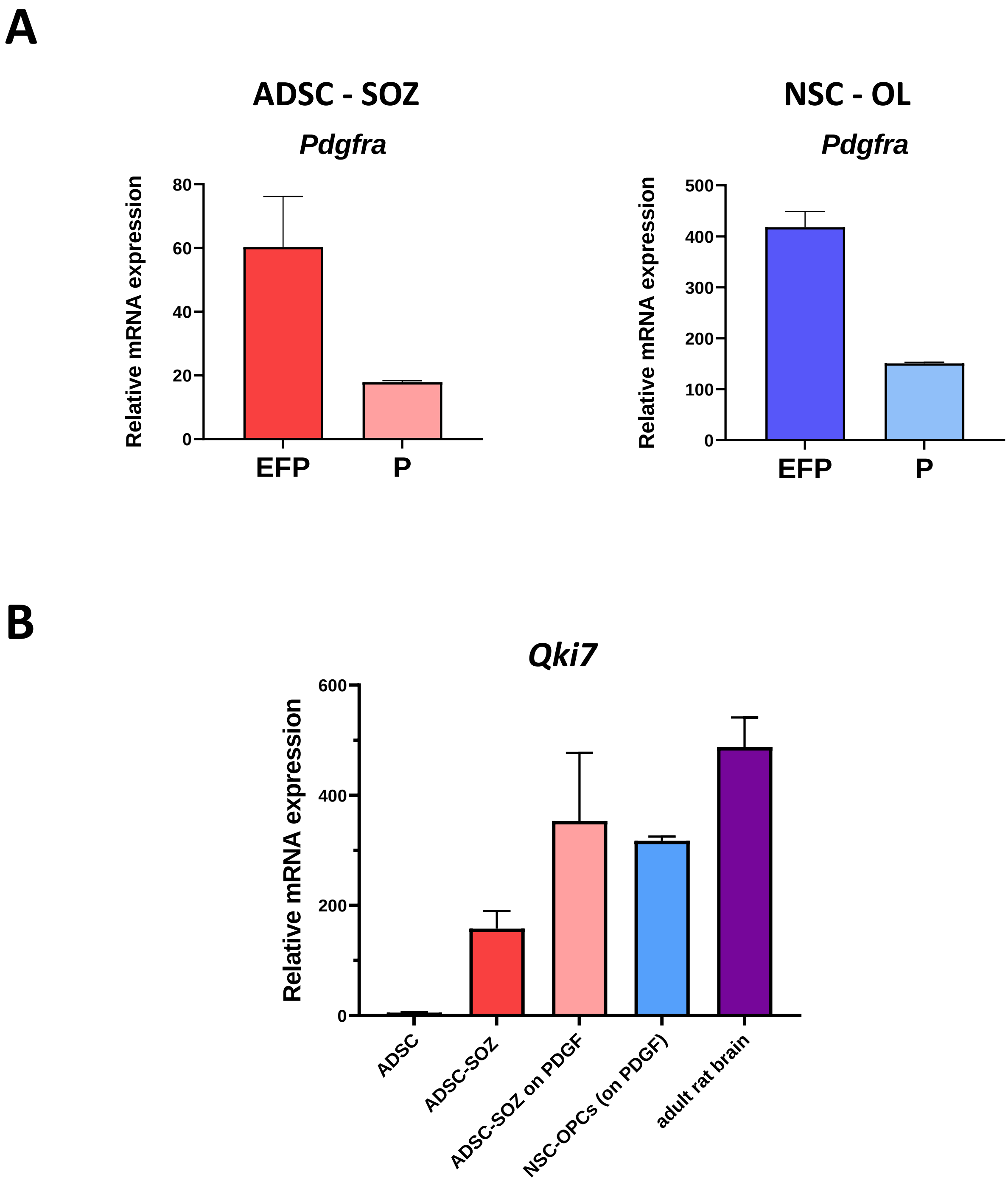

Supplementary Figure 7 **A**: Similarity of the pattern of PDGF receptor alpha (*Pdgfra*) mRNA expression in rat S + O + Z-transduced ADSCs (ADSC-SOZ, sibling cultures of one sample) and in oligodendroglia generated from rat oligospheres (NSC-OL, sibling cultures of one sample) when maintained in NBB27 + EGF + bFGF + PDGF-AA (EFP) or after 7 days in NBB27 + PDGF-AA (P). **B**: Demonstration of the variable expression of *Qki7* mRNA in different cell types and/or conditions with the primers used in this study. Real-time RT-PCR measurements of mRNA of each cell type relative to their respective GAPDH × 1000

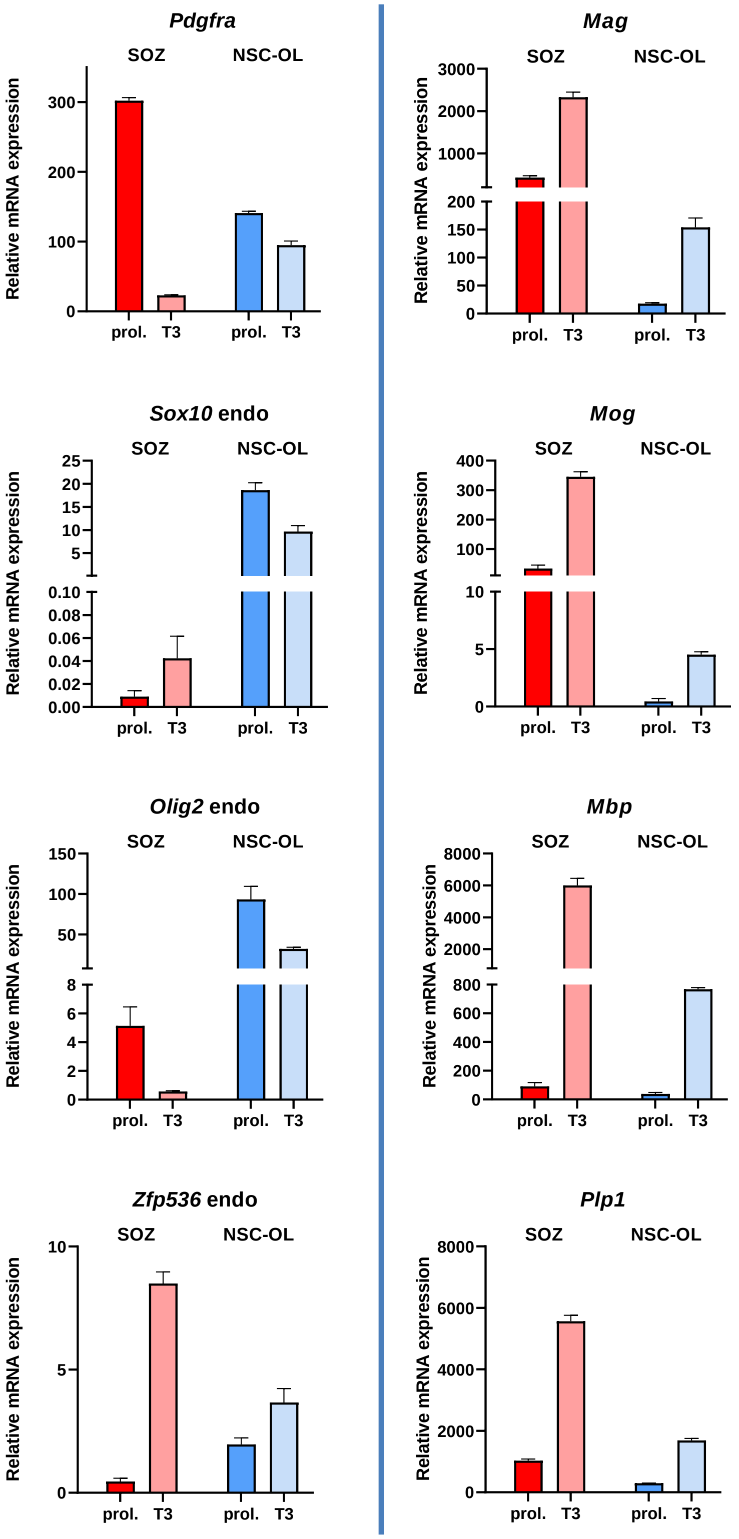

Supplementary Figure 8 **Effect of T_3 supplementation to the culture medium (NBB27) on the mRNA expression of various molecules related to oligodendroglial differentiation** in S + O + Z-transduced adult rat **ADSCs** (**SOZ**) and in cells derived from rat neural oligospheres (**NSC-OL**). **prol**: cells proliferating in NBB27 + EGF + bFGF + PDGF-AA (SOZ) or NBB27 + PDGF-AA (NSC-OL); **T3**: NBB27 + T_3. SOZ medium is additionally supplemented with doxycycline. It can be observed that both cell types, brain-derived (NSC-OL) and converted (SOZ), follow similar variation in the expression of oligodendroglial genes. RT-qPCR measurements of one example of sibling cultures of SOZ or neural oligosphere-derived cultures relative to their respective GAPDH × 1000

Role of continuous transgene expresion on the phenotype of converted cells

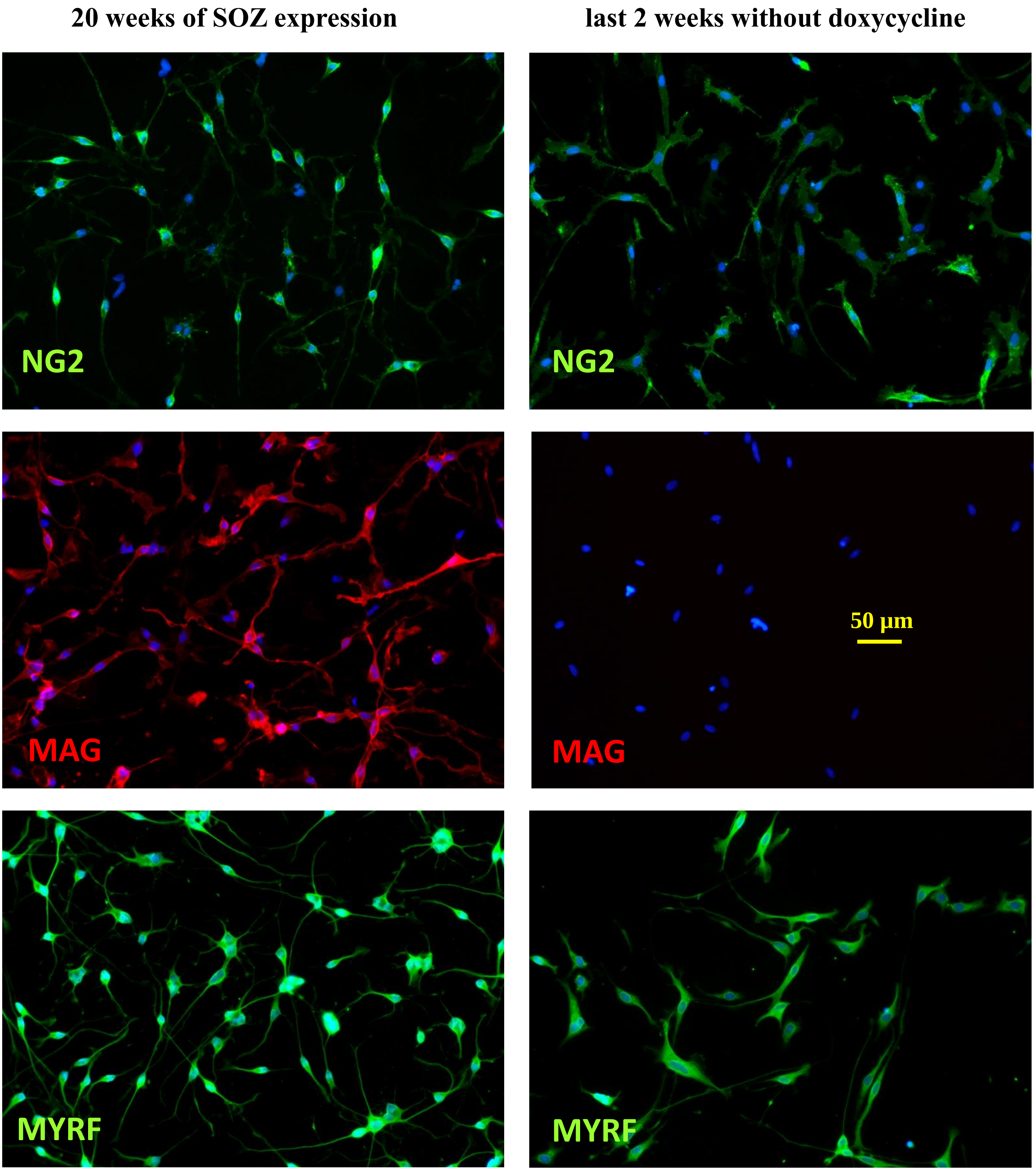

Supplementary Figure 9 Continuous S + O + Z overexpression is required for maintaining the glial phenotype. In these example images, to the left, doxycycline-induced transgene overexpression was maintained for 20 weeks. Numerous cells were $NG2^+$ and/or MAG^+, and most cells stained for MYRF. Sibling cultures that were maintained for the last 2 weeks without doxycycline lowered their proliferation, changed to a flat morphology and became MAG-negative (they were also O4-negative, not shown here). However, these "de-converted" cells kept NG2 labelling – which was already shown by ADSC before conversion- and MYRF labelling. Scale: 50 µm for all images

Differentiation by withdrawal of growth factors from the medium for 10 days

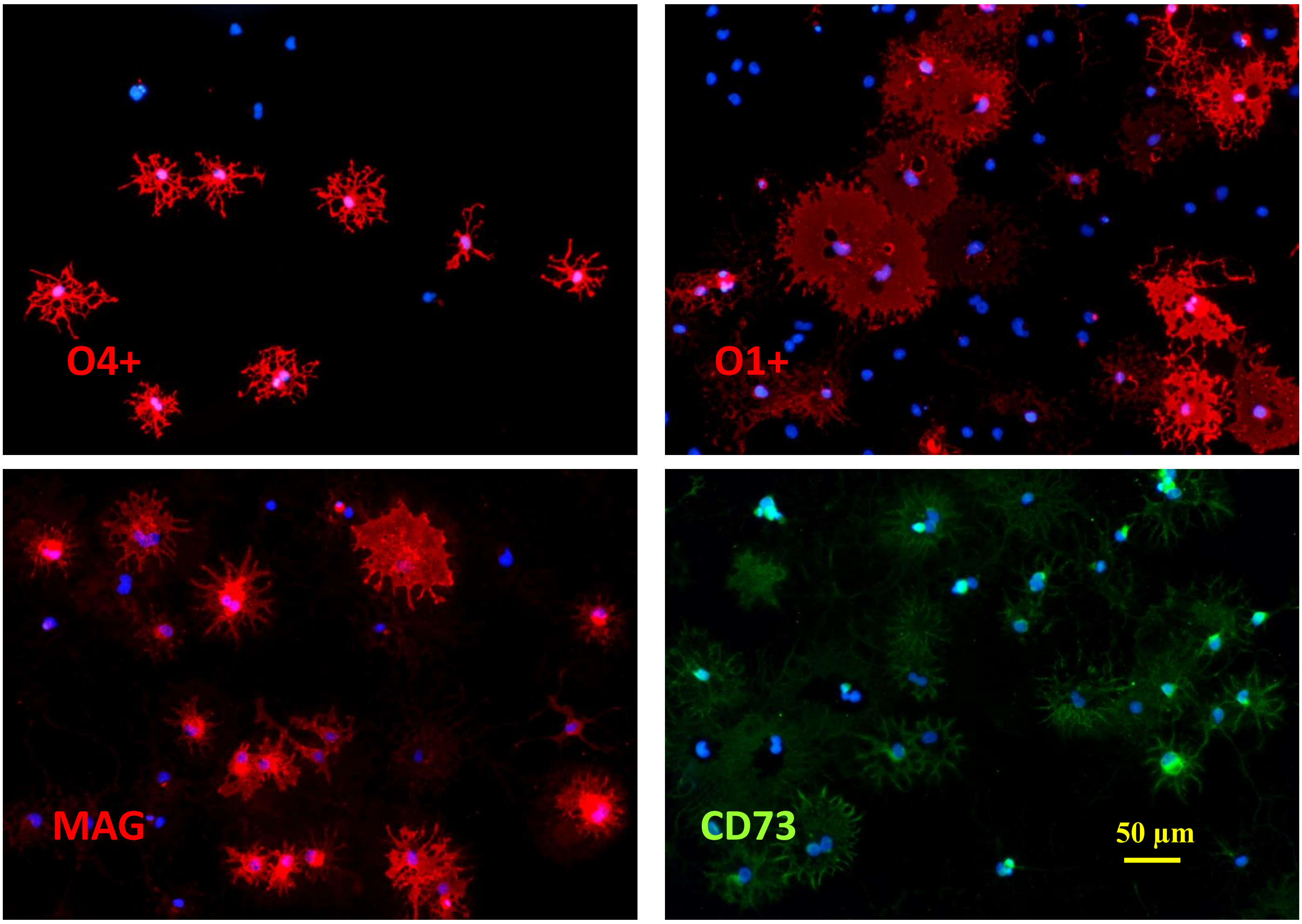

5 months of continuous proliferation **last 10 days with no growth factor in medium**

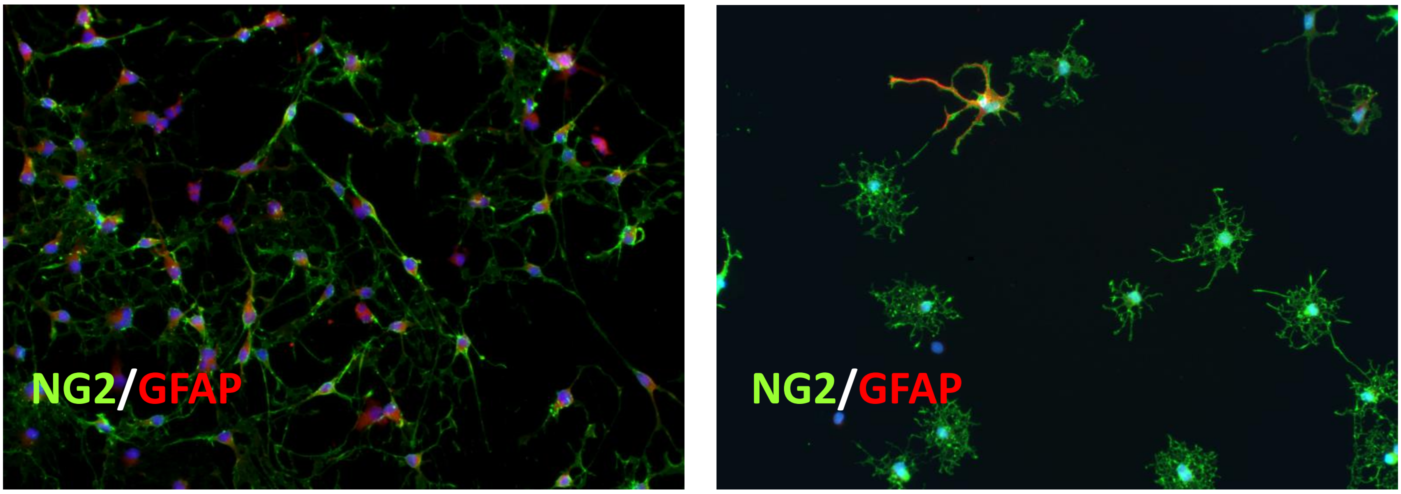

Supplementary Figure 10 **S + O + Z-converted cells can be differentiated by withdrawal of growth factors (EGF, bFGF and PDGF-AA) from the culture medium**. After 10 days with no growth factors, the cells have slowed their proliferation and increased their size. Converted cells maintained O4 and MAG labelling and started to show O1 labelling. In these conditions, cells still maintain mesenchymal-characteristic CD73 expression. In lower pictures, background labelling for GFAP (in red) is shown by most converted cells after 5 months of continuous proliferation but after 10 days in culture with no growth factor supplementation some cells start to show intense GFAP labelling while the remaining cells are totally negative. Scale: 50 µm for all images

Differentiation by culturing in DMEM + 10 % FBS + doxycycline

proliferating<br>NBB27 + EGF + bFGF + PDGF + doxy	differentiated<br>DMEM + 10% FBS + doxy (7 days)

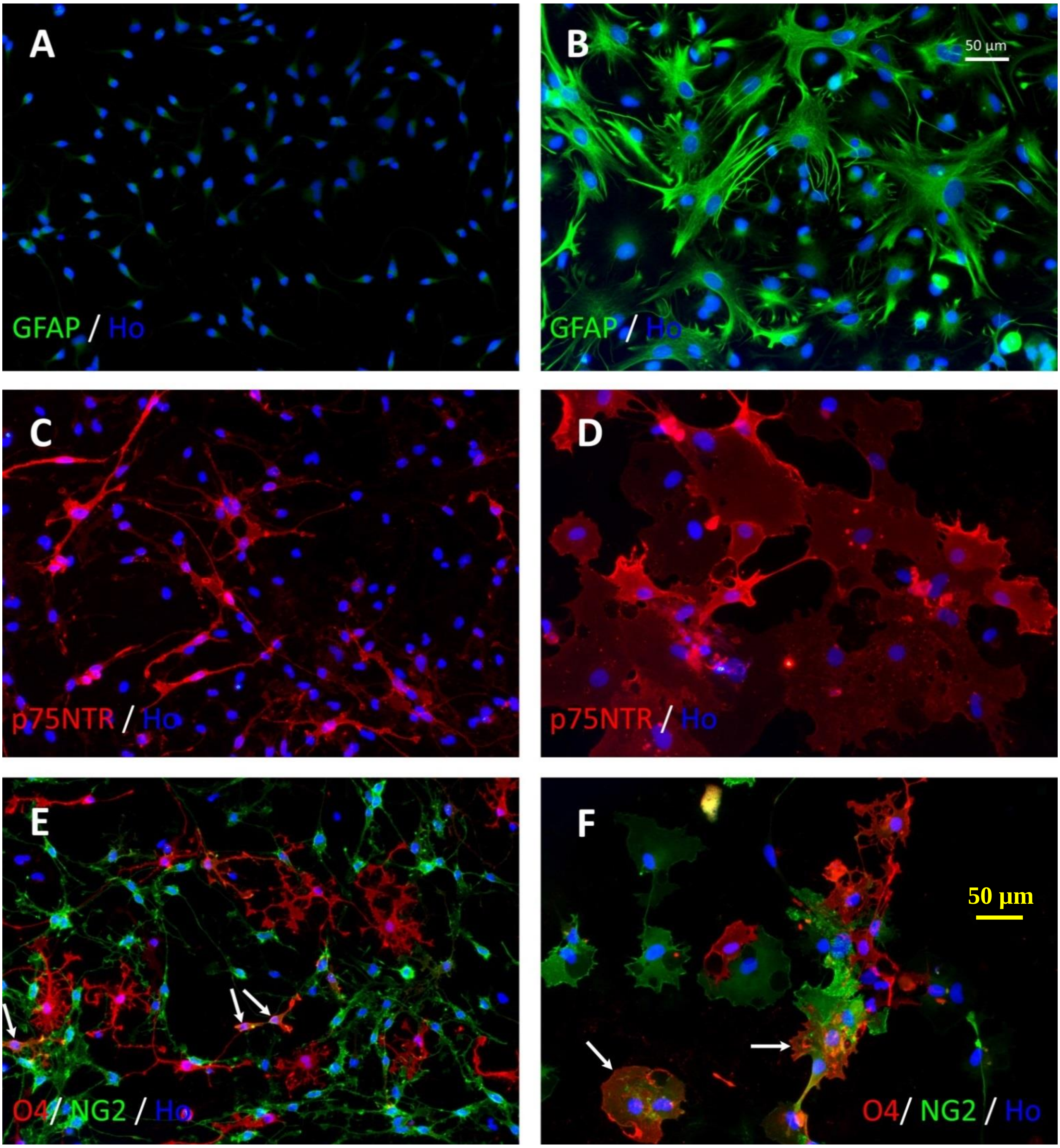

Supplementary Figure 11 **S + O + Z-converted cells can be differentiated by culturing in 10% fetal bovine serum- containing medium (D10)**. To the left (**A**, **C** and **E**) converted cells maintained in proliferating conditions (NBB27 + EGF + bFGF + PDGF-AA + doxycycline). To the right (**B**, **D** and **F**) converted cells kept for the last 7 days in D10. Serum-containing medium caused a slowed proliferation and cell enlargement and induced the differentiation of GFAP^{+} cells with the typical morphology of protoplasmic astrocytes (**B**), but also numerous flat and large p75NTR^{+} cells (**D**). O4 and NG2 markers continue being expressed by cells maintained in D10 (**F**) in proportions that are similar to those maintained in proliferating conditions (**E**) . Scale: 50 µm for all images

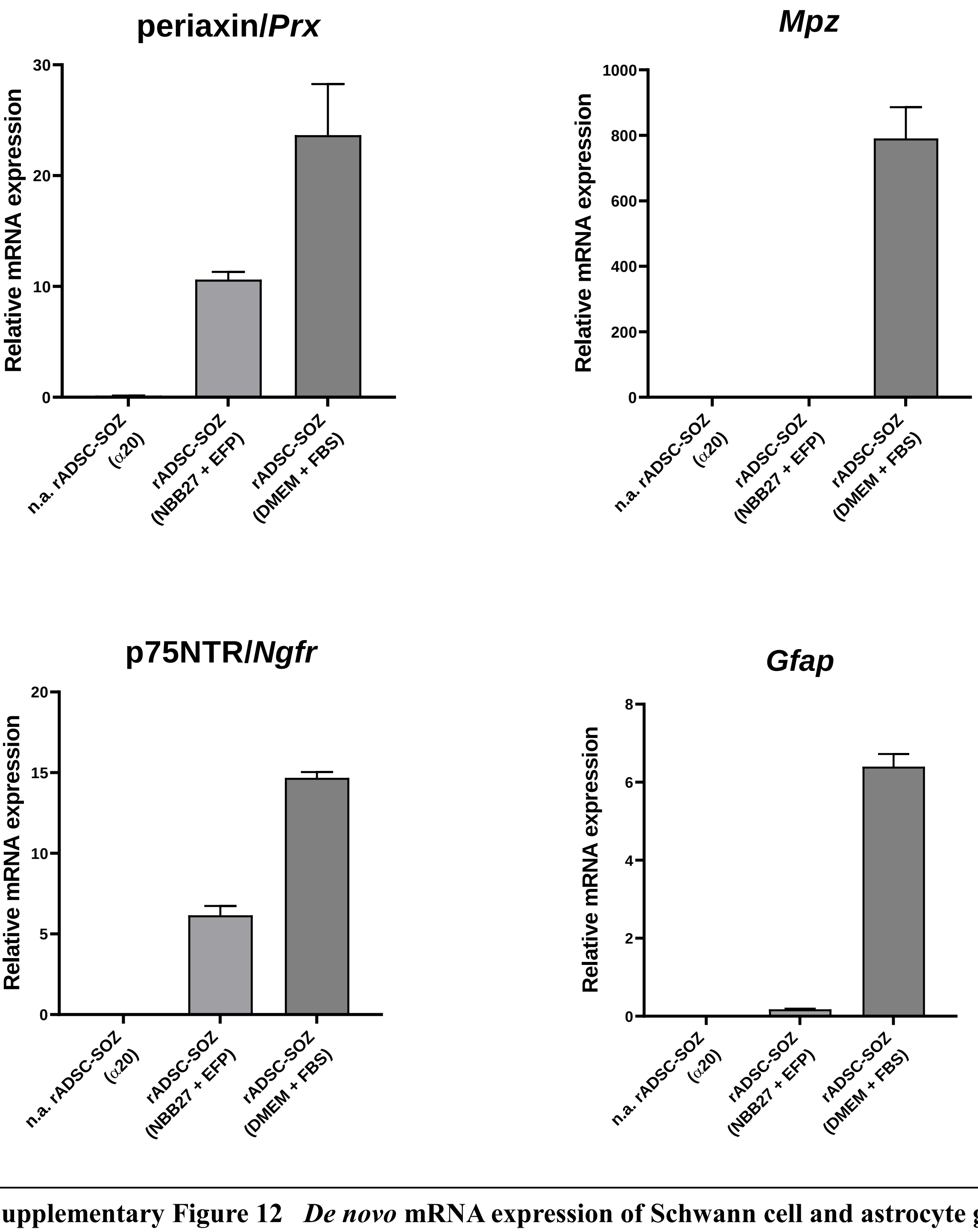

Supplementary Figure 12 ***De novo*** **mRNA expression of Schwann cell and astrocyte genes in S + O + Z-transduced adult rat ADSCs.** (rADSC-SOZ). By RT-qPCR, Periaxin (gene *Prx*), a protein expressed by developing and myelinating Schwann cells, is only detected in converted cells but not in non-activated cells (i.e, the trensgene expression is not induced with doxycycline; n.a. rADSC-SOZ). and its levels are increased when these cells are cultured in FBS-supplemented medium. Myelin protein zero (*Mpz*), a component of Schwann cell myelin, is only detected (at high levels) when converted cells are maintained in FBS-supplemented medium. Expression of p75NTR mRNA (gene *Ngfr*), characteristic of non-myelinating Schwann cells, is shown only in rADSC-SOZ and enhanced when these cells are maintained in FBS-supplemented medium. Glial filament acidic protein rRNA, *Gfap,* is expressed at low levels in rADSC-SOZ but levels are abruptly increased in cells maintained in FBS-supplemented medium. Graphs represent mRNA expression of each gene relative to *Gapdh*

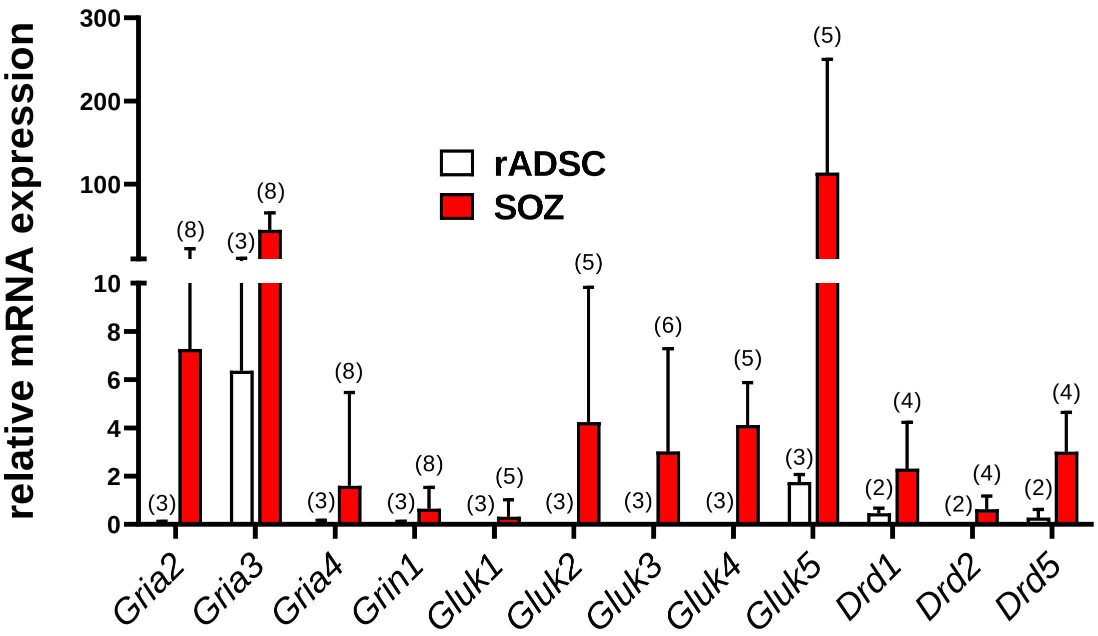

Supplementary Figure 13: RT-qPCR analysis of mRNA expression of neurotransmitter receptors in rat ADSC vs. ADSC-SOZ. This image complements Fig. 7 of the main text by extracting the data of basal ADSC (rADSC) and S + O + Z-transduced ADSC (SOZ) to compare the expression of those neurotransmitter receptors. Sample numbers, each one with technical triplicates, are indicated on top of columns. A Wilcoxon *t*-test, paring the means of each neurotransmitter receptor in rADSC *vs* SOZ, confirmed that S + O + Z-transduction in adult rat ADSC significantly increased the expression of their mRNA set ($p > 0.0001$).

neural–derived oligosphere cells (NSC-OL)

in NBB27 + EGF + bFGF + PDGF-AA (NSC-OPC)

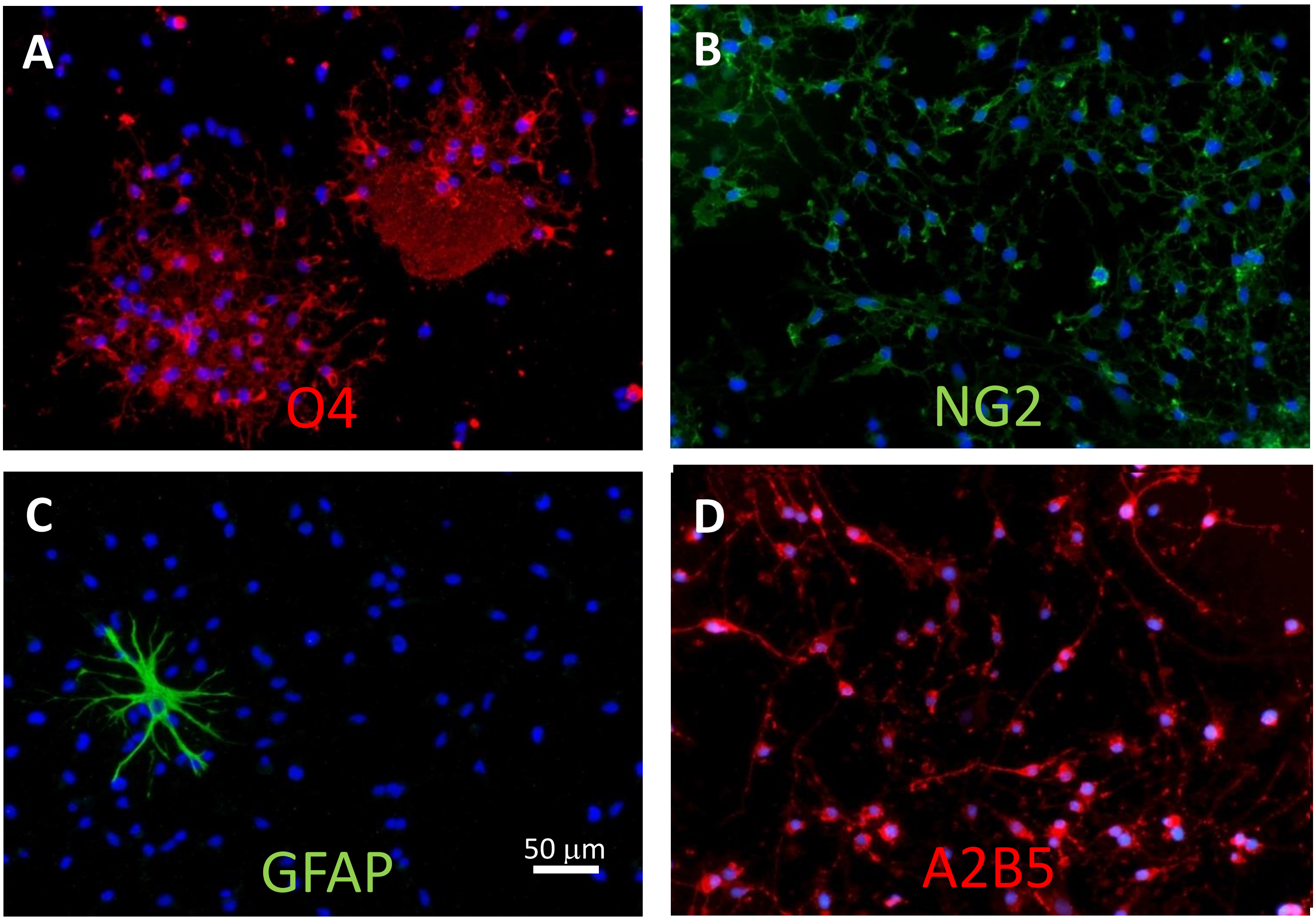

last 7 days in NBB27 + T_3 (NSC-oligodendrocytes)

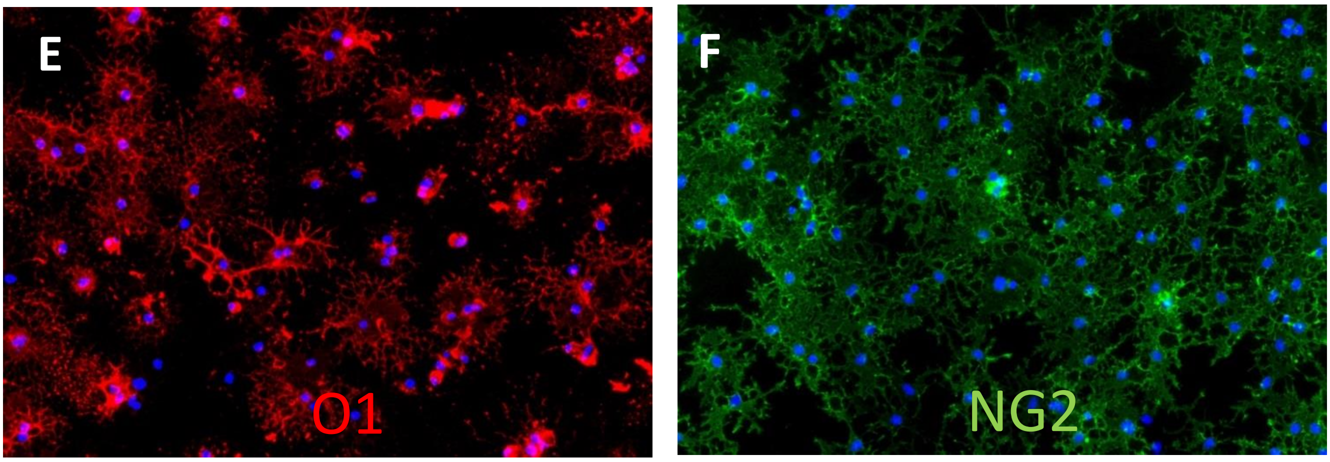

Supplementary Figure 14 Immunophenotyping of the cell composition of neural-derived oligospheres. For immunofluorescence analysis, disaggregated cells were seeded on poly-L-ornithine-coated coverslips and maintained for 7 days in the growth medium (NBB27 + EGF + bFGF + PDGF-AA, images **A-D**) or in differentiating medium, (NBB27 + T_3, images **E** and **F**). Cell counts in growth medium revealed that > 90 % of cells were $NG2^+$ or A2B5 and that $O4^+$ cells ranged between 58 – 95 % of all cells in the culture. $GFAP^+$ astrocytes made up a minor population, rarely exceeding 1 % of cells. Because of its enrichment in OPCs, we considered this culture as NSC-OPC. Maintenance of cells in differentiating conditions further demonstrated the oligodendroglial enrichment of oligospheres, since > 90 % of cells were $O1^+$ (image **E**), which indicated that OPCs differentiated to mature oligodendrocytes. Similar numbers of $NG2^+$ were observed in this condition (image **F**), although they showed more profuse branching and their reactivity was more intense.

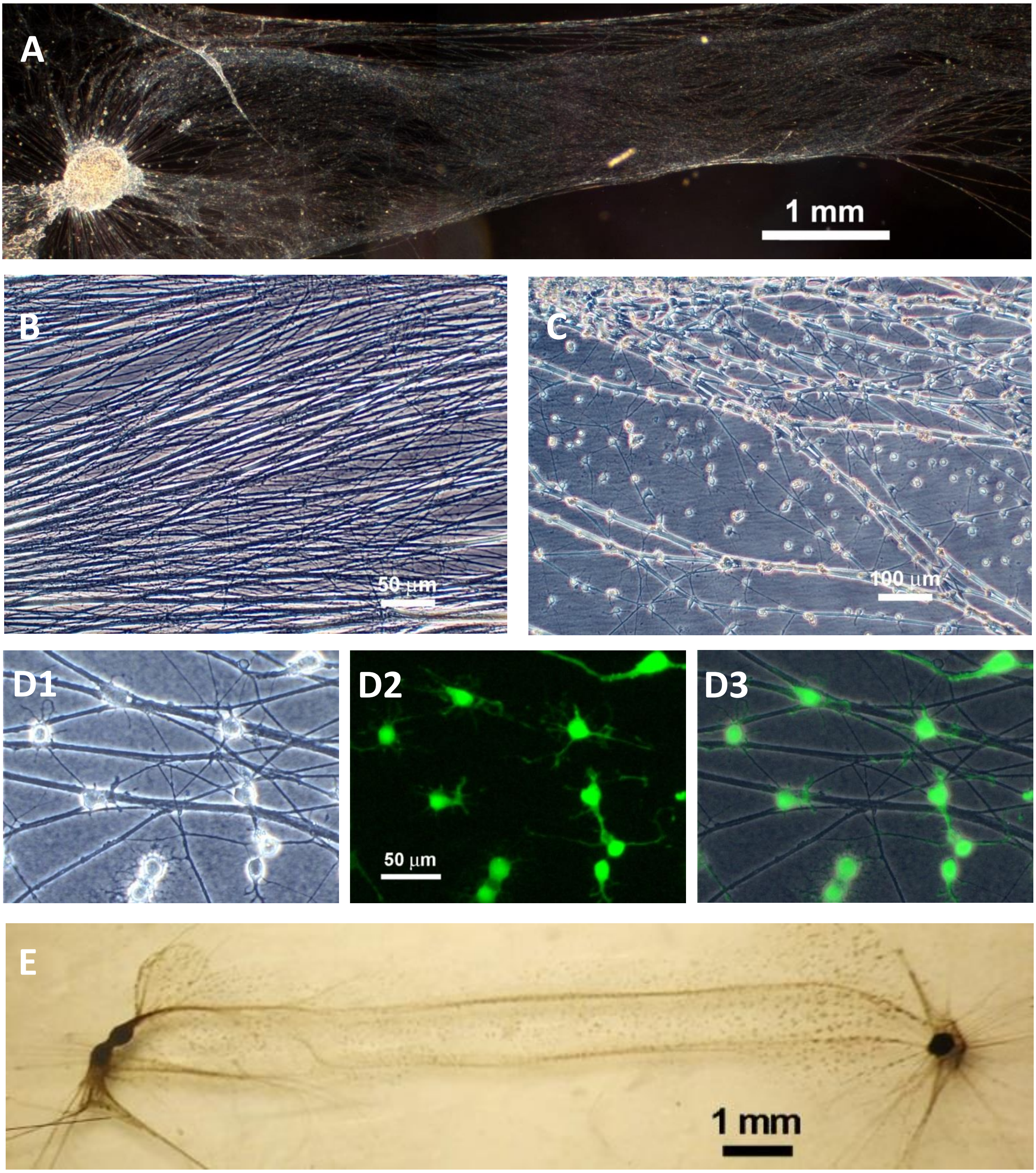

Supplementary Figure 15 Setup of co-cultures of DRG neurons with S + O + Z-transduced cells for electron microscopy. **A**: Dark field image at low magnification of one DRG whose neurons extend their axons longitudinally along a track of Geltrex. **B**: Phase contrast of parallel nude axons in the track. **C**: Shortly after seeding S + O + Z-transduced cells over the track, these cells attach or contact preferentially to axons. **D1-D3**: In a living culture, eGFP –expressing converted cells show their close contacts with axons (D1, phase contrast; D2, eGFP; D3, both D1 and D2 images combined. **E**: OsO4 fixed and stained track of axons originating from DRGs placed at each end of a Geltrex track and the converted cells that were seeded on them. The culture has been dehydrated and included in thin layer of epoxy resin for electron microscopy processing; afterward, a small piece or the axonal track will be cropped and sectioned in ultramicrotome.

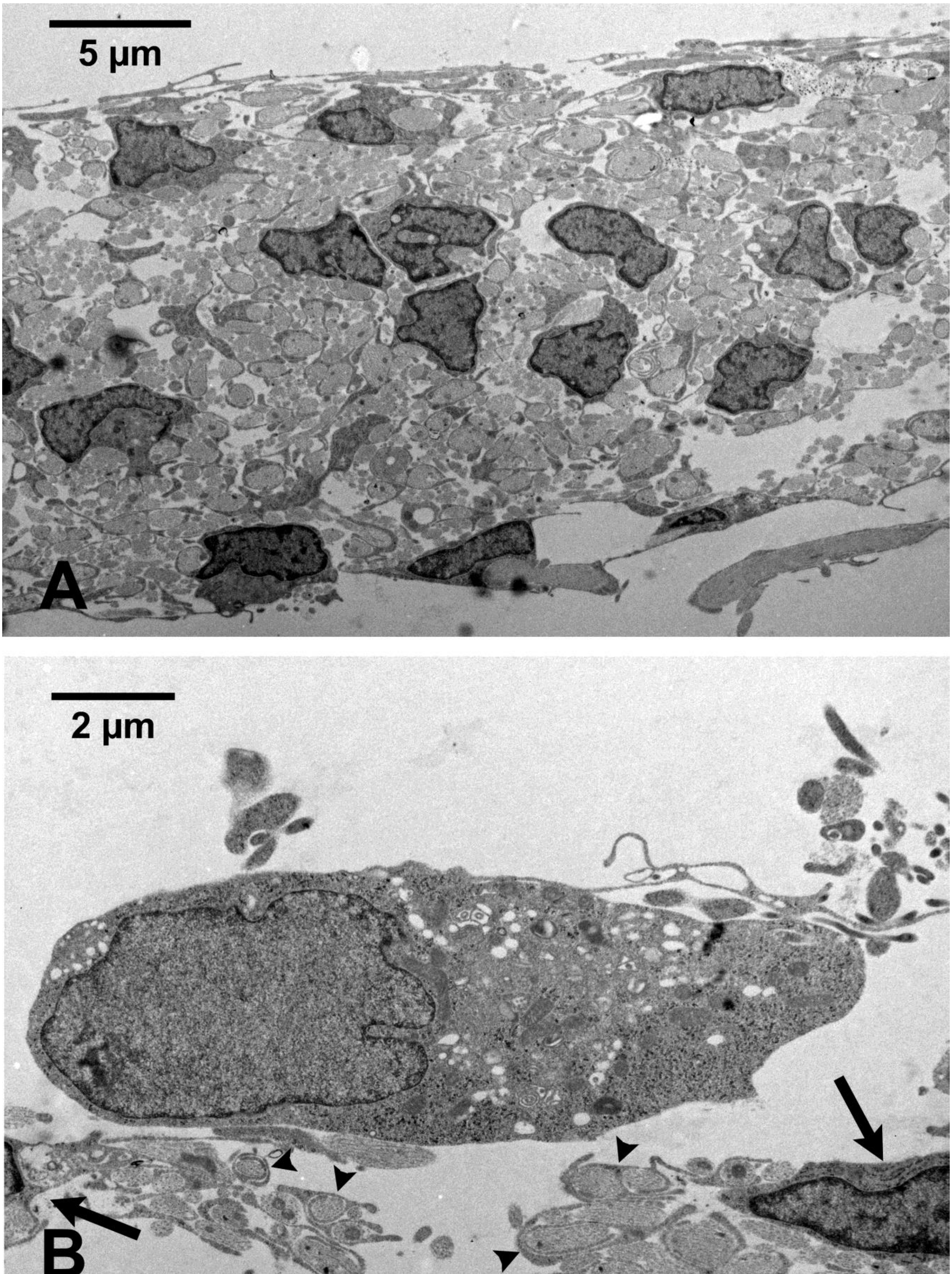

Supplementary Figure 16 **Transmission Electron Microscopy of S + O + Z-transduced cells in co-culture with DRG neurons**. **A**: Numerous oligodendroglial-like cells are found interspersed in axon bundles. **B**: Some cells of larger size and less electron-dense than oligodendroglial-like cells (arrows) are also found. They extend cytoplasmic processes but do not envelope axons as oligodendroglial-like cell do (arrowheads)

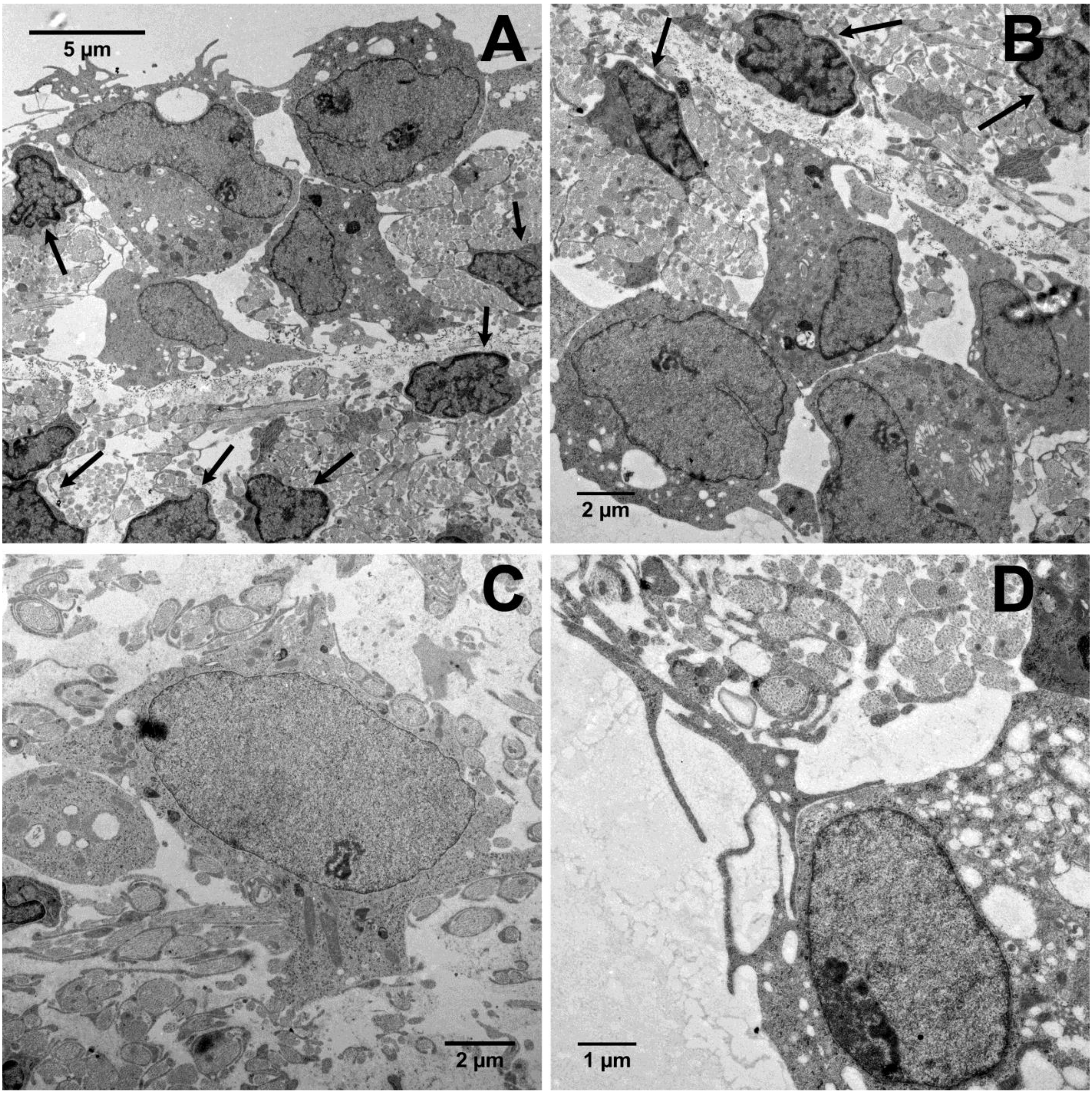

Supplementary Figure 17 Transmission Electron Microscopy of S + O + Z-transduced cells in co-culture with DRG neurons: Non-oligodendroglial cells. **A** and **B**: Non oligodendroglial cells can be found at the surface of axonal bundles, often in contact with oligodendroglial-like cells (arrows). Their appearance is also very characteristic: Of larger size and paler than oligodendroglia, their nuclei are also larger, rounder but sometimes indented, and show a rim of heterochromatin. Their cytoplasms show numerous electro-lucent droplets and ribosomes and often extend processes that envelope groups of axons and their oligodendroglial sheaths. **C** and **D**: Other examples of non-oligodendroglial cells.

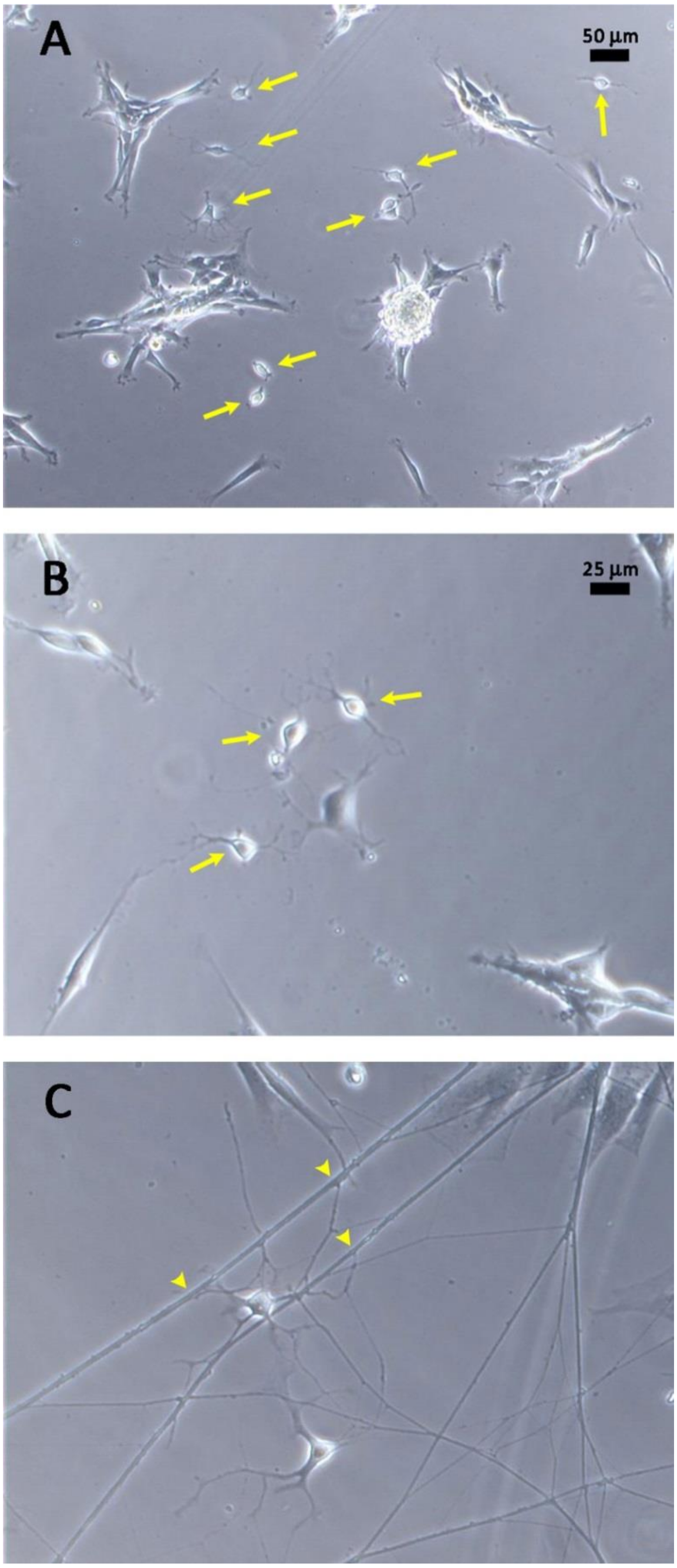

<u>Supplementary Figure 18</u> Oligodendroglial-like cells generated by expression of Sox10 + Olig2 + Nkx6.1 transgenes in adult human ADSCs maintained in NBB27 medium supplemented with EGF + bFGF + PDGF-AA. After 5 months of continuous transgene overexpression, small, refringent, $O4^{+}$ cells with short ramified processes were obtained. A and B: yellow arrows point to these cells, mixed with mesenchymal-type cells. In C these cells, when co-cultured with DRGn attach to axons (arrowheads). Scale: 50 µm in A and 25 µM in B and C. Culture procedures for human ADSCs were approved by the Ethics Committee for Clinical Research of the Ramón y Cajal Hospital.

Supplementary Table 1. Commercial references of products used in the present work

Product	Supplier	Cat nr.
collagenase A	Roche	10103578001
αMEM (Minimal Essential Medium Eagle, alpha modification)	Sigma-Aldrich	M4526
FBS (foetal bovine serum)	Gibco	26140079
MEM non-essential amino acids	Gibco	11140050
glutamine	Gibco	25030024
antibiotic/antimitotic	Gibco	15240062
DNAse I	Roche Applied Science	1284932
100 µm mesh cell strainers	BD Biosciences	43-57100-51
75 cm^2 flask for MSCs	Falcon	353135
Hank's balanced salt solution, HBSS	Capricorn	HBSS-3A
0.5% trypsin + 0.2 % EDTA (10x)	Sigma-Aldrich	T4174
Lipofectamine 2000	Invitrogen	11668030
Dulbecco's minimal essential medium, DMEM	Biowest	L0101
0.45 µm filters Millex-HA	Millipore	SLHA033SS
qPCR Lentivirus titer kit	Applied Biological Materials	LV900
Lightcycler 480 SybrGreen I Master kit	Roche Applied Science	04707516001
polybrene	Sigma-Aldrich	H9268
DMSO, sterile filtered	Sigma-Aldrich	D2650
75 cm^2 or 25 cm^2 flasks for OPC and NSC culture	Nunc	156472 or 156340
Neurobasal medium	Invitrogen	21103049
B27 supplement	Invitrogen	08-0085SA
doxycycline hydrochloride	Fisher Scientific	BP2653
animal-free recombinant human EGF	PeproTech	AF-100-15
recombinant human FGF-basic	PeproTech	100-18B
recombinant human PDGF-AA	Immunotools	11343685
recombinant human β-NGF	PeproTech	450-01
d-biotin	Sigma-Aldrich	B4639
all-trans retinoic acid	Sigma-Aldrich	R2625
Repsox	Selleckchem	S-7223
insulin	Sigma-Aldrich	I1882
indomethacin	Sigma-Aldrich	I7378
3-isobutyl-1-methylxanthine, IBMX	Sigma-Aldrich	I7018
poly-L-ornithine	Sigma-Aldrich	P3655
12 mm∅ round glass coverslips	Marienfeld	0111520
24-well plates	Costar	3524
PBS tablets	Gibco	18912014
bis-benzimide hydrochloride, Hoechst 33342	Sigma-Aldrich	B2261
Prolong Gold mounting medium	Molecular Probes	P36930
RNeasy Mini kit	Qiagen	74004
GeneJET RNA purification kit	Thermo Scientific	K0731
NZY First-strand cDNA synthesis kit	NZYTech	MB125
dNTP mix, 10 nM each	Fisher Scientific	R0191
AmpliTaq DNA polymerase	Applied Biosystems	N8080160
Agarose LE	Promega	V3125
GreenSafe Premium	NZYTech	MB13201
TB Green^{TM} Premix Ex Taq^{TM}	Takara Bio	RR420B
StemPro Accutase	Gibco	A1110501
Accutase	Sigma-Aldrich	A6964
bovine serum albumin, BSA	Sigma-Aldrich	A8806
anti-mouse IgG Microbeads	Miltenyi	130-048-401
laminin	Sigma-Aldrich	L2020
fluorodeoxyuridine	Alfa Aesar	L16497
uridine	Sigma-Aldrich	U6381
benztropine mesylate	Santa Cruz	sc-202495
Petri dishes, ∅35 mm	Nunc	153066
collagen type I solution from rat tail	Sigma-Aldrich	C3867
Geltrex (Reduced Growth Factor Basement Membrane Matrix)	Gibco	A1413201
glutaraldehyde 25% solution	Electron Microscope Sciences	16210
OsO4 4% solution	Electron Microscope Sciences	19150
Epoxy-embedding medium kit	Sigma-Aldrich	45359
T_3 (3,3′,5-Triiodo-L-thyronine sodium salt)	Sigma-Aldrich	T6397

Supplementary Table 2

Phase-contrast microscopy observations on the effect of different transcription factor combinations together with pre-treatments before transgene induction with doxycycline, on the appearance of cells with oligodendroglial morphology (small, round, refringent body. with few branches). S: Sox10; O: Olig2; Z: Zfp536; N: Nkx6.1. Pre-treatments are indicated as an additional letter: a: adipose cocktail; R: retinoic acid; X: Repsox.

Please, note that appearance of cells with oligodendroglial morphology does not demonstrate O4 labelling neither oligodendroglial conversion. ***** Indicates that, due to a gradual morphological change, timing for first oligodendroglial-like cells is not precise

**Transcription factor combination + pre-treatment**	**Time after doxycycline induction for appearance of a first cell with oligodendroglial morphology**	**Additional time for showing up colonies of 4 independent cells**
**SOZ_a**	2-3 weeks*	1-1 ½ weeks
**SOZ_R**	4 weeks	2 weeks
**SOZ_X**	4 weeks	2 weeks
**SOZ_{R+X}**	4 weeks	2 weeks
**SOZ_{a+X}**	4 weeks	2 weeks
**SON_a**	Negative	
**$SONZ_a$**	3 weeks	1 week
**$SONZ_R$**	5 weeks	1 week
**OZa**	8 weeks	Negative
**OZ_R**	5 weeks	2 weeks
**OZ_X**	8 weeks	Negative
**OZ_{R+X}**	8 weeks	Negative
**O_a**	6 weeks	Negative
**O_R**	6 weeks	1 week
**O_X**	8 weeks	Negative
**O_{R+X}**	8 weeks	Negative

Supplementary Table 3
Primer combinations used for characterization in the present study for real-time PCR (qPCR, asterisks) or only for end-point PCR

mRNA	primers	amplicon	qPCR
Sox10<br>(endogenous)	5′- GGCACGCAGAAAGTTAGCCGA -3′<br>5′- GGGCGCTTGTCACTCTCGTTCA -3′	105 bp	*
Olig2<br>(endogenous)	5′- GCAGCGAGCACCTCAAATCG -3′<br>5′- ATGGCCCCGGAGACGATCTA -3′	85 bp	*
Zfp536<br>(endogenous)	5′- TCTGGCCGTCTTTCAGCATC -3′<br>5′- CACTCCAAGGCACAGACTCG -3′	127 bp	*
Cnp	5′- AGGCGTGCTGCACTGTACAACC -3′<br>5′- TGGACAGTTTGAAGGCCTTGCCG -3′	116 bp	
Mbp total<br>(non-Golli)	5′- AGAACTACCCACTACGGCTCCCT -3′<br>5′- TTGGGATGGAGGGGGTGTACGA -3′	114 bp	*
Mbp-5<br>(distinguishes 5<br>non-Golli<br>variants)	5′- TCCTTGACTCCATCGGGCGCT -3′<br>5′- GGAGATCCAGAGCGGCTGTCT -3′	460 bp (variant 1)<br>382 bp (variant 2)<br>337 bp (variant 3)<br>349 bp (variant 4)<br>259 bp (variant 5)	
Mag total	5′- AGGACGATGGGGAGTACTGG -3′<br>5′- GCACAGTGCGATTCCAGAAG -3′	112 bp	*
S-Mag	5′- GGGAGACAACCCTCATGTCC -3′<br>5′- TGGGGCTCTCAGTGACAATC -3′	108 bp	
L-Mag	5′- GCTACATCACCCAGACAAGAAGA -3′<br>5′- CAGGCGCTTCTCACTCTCATACT -3′	140 bp	
Mog	5′- TGCAGCCAGAGGGCCTTAGCTT -3′<br>5-′ ATTCAGGCGCTTGCTCTGCGT -3′	341 bp (w/ signal peptide)<br>260 bp (w/o signal peptide)	
Mog	5′- TAAAGATGGCCGGTGTGTGGA -3′<br>5-′ CCCTGGCCCTATCACTCTGAA -3′	113 bp	*
Plp1/Dm20	5′-GGCCGAGGGCTTCTACACCAC-3′<br>5′-CAGGAGCCCACTGTGGAGCAA- 3′	792 bp (PLP1 variant)<br>687 bp (DM20 variant)	
Plp1	5′- TTTGGAGCGGGTGTGTCATT -3′<br>5′- GGCAAACACCAGGAGCCATA - 3′	103 bp	*
Gfap	5′- AGAAAACCGCATCACCATTC -3′<br>5′- TCCTTAATGACCTCGCCATC -3′	150 bp	*
Qki-7	5′- CTACACGTTGGCACCAGCTA -3′<br>5′- TCAGGCATGACTGGCATTTCA -3′	90 bp	*
Gjc2<br>(connexin 47)	5′- GTGGAAGGGCTCATCAGAAGG -3′<br>5′- TTGCCCACGAAGGTGGAATG -3′	137 bp	*
Gja1<br>(connexin 43)	5′- AGGAGTTCCACCAACTTTGGC -3′<br>5′- GTGGAGTAGGCTTGGACCTTG -3′	120 bp	*
Gjc3<br>(connexin 29)	5′- GGTTGATGCTTCTGCTTGGAG -3′<br>5′- AGACGGAATCCCACGAGCA -3	137 bp	*
Gjb6<br>(connexin 30)	5′- GTGGTTCTTGGACTGGACGAC -3′<br>5′- CCATACCTTCCCTATGCTGGTC -3′	134 bp	*
Pdgfra<br>(PDGFRα)	5′- AAGATGCTCAAACCCACAGC -3′<br>5′- ACAATGTTCAGATGCGGTCC -3′	95 bp	*
Prx<br>(Periaxin)	5′- GGAATCTTTGTCCGCGAGCTG -3′<br>5′- CTCAGAAGTTGGTCCCCTTCCT -3′	80 bp	*
Mpz (Myelin<br>protein zero)	5′- CTCTTCTCTTCTTTGGTGCTGTC -3′<br>5′- TCTGAGACCCATTCACTGGAC -3′	128 bp	*
Gapdh<br>(housekeeping)	5′- AGTGCCAGCCTCGTCTCATAGA -3′<br>5′- CACAAGAGAAGGCAGCCCTGGT -3′	96 bp	*

Supplementary Table 4.
Primer combinations used for rat neurotransmitter receptors in real-time PCR (qPCR)

mRNA	primers	amplicon	NCBI Reference Sequence
Gria2 (AMPA-R2)	5'- CAGCATACAGATAGGGGGGCTA -3'<br>5'- TCCGAAGTGGAAAACTGAACCA -3'	90 bp	NM_017261.2
Gria3 (AMPA-R3)	5'- GGCTACAACGTGTATGGAACAG -3'<br>5'- CACCAGGGAGAGTGAAATCCG -3'	132 bp	NM_032990.2
Gria4 (AMPA-R4)	5'- AGAAAACCTGGGCGATCTGC -3'<br>5'- GACAATCTGCCTGCAAATAATCCTC - 3'	135 bp	NM_017263.2
Grin1 (NMDA-R1)	5'- CCCGAATGTCCATCTACTCTGAC -3'<br>5'- GGTTCCAGTTGTAGACTCGCA -3'	116 bp	NM_001270602.1
Grik1 (kainate receptor subunit GluK1)	5'- CAGTGCCATCGACATAAGCCAT -3'<br>5'- GCCATCGGTCTTATTGAAGGTGA -3'	111 bp	NM_001111117.1
Grik2 (kainate receptor subunit GluK2)	5'- TCGCTCCATTAAAGTCCTGCT -3'<br>5'- CATGGGGCCAGATTCCACAT -3'	109 bp	NM_019309.2
Grik3 (kainate receptor subunit GluK3)	5'- ACTCCCTACAGTGCCATCGG -3'<br>5'- AATCCGTCCAGTTAATCCTTCCC -3'	98 bp	NM_001112716.1
Grik4 (kainate receptor subunit GluK4)	5'- CATGGAAGTGCCCATCGAGT -3'<br>5'- ATGCGCTGGTAGGTCTGGT -3'	126 bp	NM_012572.2
Grik5 (kainate receptor subunit GluK5)	5'- ACAGCCAGTACGAGACTACGG -3'<br>5-' TCTCCCCACAGATATGGCTCAC -3'	120 bp	NM_031508.2
Drd1 (dopamine R1)	5'- GCGTAGCATGGACTCTGTCT -3'<br>5-' CGTCCTGCTCAACCTTGTGT -3'	149 bp	NM_012546.3
Drd2 (dopamine R2)	5′- CCAACCTGAAGACACCACTCA -3′<br>5′- ATCCATTCTCCGCCTGTTCAC - 3′	113 bp	NM_012547.1
Drd5 (dopamine R5)	5′- ATGGGGGTCTTCGTGTGTTG -3′<br>5′- GTCTCGCTGACACAAGGGAA - 3′	116 bp	NM_012768.1

Supplementary Table 5

Primary antibodies used in the study and their source

Antigen	Antibody	Host	Dilution ICC
MOG	Santa Cruz SC-73330	mouse (clone NYRMOG)	1:100
MBP	Abcam AB7439 and Millipore MAB386	rat (clone 12)	1:100
PLP1	Invitrogen MA-1-80034	mouse (clone PLPC1)	1:50
CNPase	Santa Cruz SC-30158	rabbit	1:100
Olig2	GenScript A01474	rabbit	1:100
Sox10	R&D Systems MAB2864	mouse	1:100
GFAP	DAKO Z334	rabbit	1:500-800
GFAP	Sigma-Aldrich G3893	mouse (clone G-A-5)	1:200
NG2 chondroitin sulphate proteoglycan	Millipore AB5320	rabbit	1:300
NG2 chondroitin sulphate proteoglycan	Sigma-Aldrich ZooMab ZRB5320	rabbit (clone 1L2)	1:100
β-tubulin isotype III	Covance PRB-435P and BioLegend 802001	rabbit	1:1000
MYRF	Cusabio CSB-PA897527LA01HU	rabbit	1:100
CD73	ProteinTech 12231	rabbit	1:200
Neurofilament 165 kDa	hybridoma supernatant (DSHB*) (Dodd et al. 1988) RRID: AB_531793	mouse (clone 2H3)	1:50
Neurofilament 210 kDa phosphorylated	hybridoma supernatant (DSHB*) (Wood and Anderton 1981) RRID: AB_528399	mouse (clone RT97)	1:10
low affinity NGF receptor (p75NTR)	hybridoma supernatant (Chandler et al. 1984)	mouse (clone 192)	1:10
MAG	hybridoma supernatant (Poltorak et al. 1987)	mouse (clone 513)	1:10
A2B5-reactive gangliosides	hybridoma supernatant (Eisenbarth et al. 1979)	mouse (clone A2B5)	1:10
oligodendroglial sulfated galactolipids	hybridoma supernatant (Sommer and Schachner 1981)	mouse (clone O4)	1:10
galactocerebroside	hybridoma supernatant (Sommer and Schachner 1981)	mouse (clone O1)	1:10

**Antibodies developed by the referred authors and obtained from the Developmental Studies Hybridoma Bank (DSHB), maintained by the University of Iowa*

References

Chandler CE, Parsons LM, Hosang M, Shooter EM. 1984. A monoclonal antibody modulates the interaction of nerve growth factor with PC12 cells. J Biol Chem 259:6882-9.

Dodd J, Morton SB, Karagogeos D, Yamamoto M, Jessell TM. 1988. Spatial regulation of axonal glycoprotein expression on subsets of embryonic spinal neurons. Neuron 1:105-16.

Eisenbarth GS, Walsh FS, Nirenberg M. 1979. Monoclonal antibody to a plasma membrane antigen of neurons. Proc Natl Acad Sci U S A 76:4913-7.

Poltorak M, Sadoul R, Keilhauer G, Landa C, Fahrig T, Schachner M. 1987. Myelin-associated glycoprotein, a member of the L2/HNK-1 family of neural cell adhesion molecules, is involved in neuron-oligodendrocyte and oligodendrocyte-oligodendrocyte interaction. J Cell Biol 105:1893-9.

Sommer I, Schachner M. 1981. Monoclonal antibodies (O1 to O4) to oligodendrocyte cell surfaces: an immunocytological study in the central nervous system. Dev Biol 83:311-327.

Wood JN, Anderton BH. 1981. Monoclonal antibodies to mammalian neurofilaments. Biosci Rep 1:263-8.
